# Are the factors associated with cognitive performance generalizable across cohorts?

**DOI:** 10.1016/j.nicl.2026.104064

**Published:** 2026-09-18

**Authors:** Peiwei Liu, Theresa M. Harrison, Heather M. Snyder, Charles DeCarli, Pauline Maillard, Prashanthi Vemuri, Danielle Harvey, Robert Koeppe, William Jagust, Laura D. Baker, Susan M. Landau, Alzheimer's Disease Neuroimaging Initiative (ADNI)¹ and the U.S. POINTER Study Group.

**Affiliations:** a Department of Neuroscience, University of California, Berkeley, CA, USA; b Medical and Scientific Relations, Alzheimer's Association, Chicago, IL, USA; c Department of Neurology, University of California, Davis, CA, USA; d Department of Radiology, Mayo Clinic, Rochester, MN, USA; e Public Health Sciences, University of California, Davis, CA, USA; f Department of Radiology, University of Michigan, Ann Arbor, MI, USA; g Gerontology and Geriatric Medicine, Wake Forest University School of Medicine, Winston-Salem, NC, USA

**Keywords:** Alzheimer's disease, Cognition, Tau PET, Aβ PET, Vascular risk, Brain atrophy

## Abstract

**Objective:**

To examine whether vascular risk and brain atrophy account for cohort differences in the associations between tau burden and cognition across cognitive domains in U.S. POINTER and ADNI.

**Methods:**

We analyzed baseline data from 775 U.S. POINTER and 405 ADNI participants without significant cognitive impairment and matched on age, sex, clinical status, and APOE ε4 status. Cognitive outcomes included global cognition, verbal memory, and executive function. Regional tau (entorhinal and meta-temporal), vascular risk biomarkers, and structural MRI measures (entorhinal cortical thickness and hippocampal volume) were examined using linear regression models stratified by amyloid status and adjusted for age, sex, education, and average number of prior cognitive test exposures. Interaction terms (biomarker × cohort) were used to assess cohort differences. False discovery rate (FDR) correction was applied.

**Results:**

Greater tau burden was associated with poorer cognitive performance across global cognition, verbal memory, and executive function in ADNI compared to U.S. POINTER, particularly among Aβ + individuals (corrected *p* < .05). Unexpectedly, vascular risk biomarkers contributed minimally to cognitive variability in either cohort. Finally, brain atrophy, particularly hippocampal volume, made a tau-independent contribution to verbal memory in ADNI but not in U.S. POINTER, especially among Aβ + individuals (corrected *p* < .05).

**Conclusions:**

Tau pathology in the presence of Aβ was a stronger predictor of cognition in ADNI than in U.S. POINTER, and this discrepancy was not explained by increased vascular risk or brain atrophy in U.S. POINTER. The drivers of cognition differed between cohorts in a way that is not easily attributable to clinical characteristics.

## Highlights

• Tau more strongly related to cognition in ADNI than U.S. POINTER among Aβ + adults.
• Vascular risk showed weak to null associations with cognition in both cohorts.
• Brain atrophy had a tau-independent role in cognition in ADNI but not U.S. POINTER.
• Cohort recruitment influences how AD biomarkers relate to cognition in older adults.

## 1 Introduction

Alzheimer's disease (AD) is pathologically defined by the accumulation of amyloid-β (Aβ) plaques and tau neurofibrillary tangles in the brain (Jack Jr. et al., 2024). Previous studies have demonstrated that Aβ deposition often begins more than a decade before the onset of clinical symptoms and may facilitate the spread of tau into neocortical regions (Jagust, 2018). Tau burden, in turn, has been shown to more directly track with neurodegeneration and cognitive decline, particularly in the entorhinal cortex and medial temporal lobe regions (Jagust, 2018).

In addition to Aβ and tau, vascular pathology is increasingly recognized as a critical contributor to age-related cognitive decline and dementia (Jagust, 2018). Epidemiological and neuroimaging evidence suggests that vascular risk biomarkers—such as non-neuroimaging cardiovascular risk factors (e.g., hypertension, obesity, smoking, and diabetes) and neuroimaging-based cerebrovascular biomarkers (e.g., white matter hyperintensity volumes and cerebral microbleeds)—may influence brain structure and function and exacerbate AD pathology (Greenberg et al., 2020; Kapasi et al., 2021). However, the extent to which vascular risk biomarkers interact with or contribute independently to cognitive decline remains unclear. One reason for this uncertainty is that individuals with cardiovascular risk factors are often excluded from observational AD neuroimaging research and clinical trials (Pittock et al., 2023). As a result, existing studies often lack adequate representation of the broader U.S. older adult population, including individuals from historically underrepresented racial and ethnic groups who also face a disproportionate burden of cardiovascular disease (Johnson et al., 2021).

The U.S. Study to PrOtect Brain Health Through Lifestyle INTErvention to Reduce risk (U.S. POINTER) was a large-scale multidomain two-year lifestyle intervention clinical trial that was designed to recruit a cohort that better reflects a more representative sample of older adults without significant impairment in the United States with respect to cardiovascular risk, ethnoracial group membership, and geographic diversity than has typically been included in AD research (Baker et al., 2024; Harrison et al., 2025; Landau et al., 2025).

In contrast, AD observational studies such as the Alzheimer's Disease Neuroimaging Initiative (ADNI) have historically limited enrollment of individuals with non-AD comorbid conditions, limiting the generalizability of findings in ADNI to real-world populations (Babulal et al., 2019; Brickman et al., 2021; Jack Jr. et al., 2024). In a recent study comparing these cohorts, we found that tau and other AD biomarkers were much less strongly associated with global cognition in U.S. POINTER imaging participants than in a matched ADNI subsample, suggesting a substantial amount of unexplained variance in cognitive performance in the community-recruited U.S. POINTER cohort (Landau et al., 2026). Here, we further investigated factors that may underlie this discrepancy by examining the roles of regional tau, regional atrophy (Ossenkoppele et al., 2019), and a variety of vascular and cerebrovascular risk measures in accounting for performance across cognitive domains (memory, executive function) in each cohort. Given the higher prevalence of vascular risk factors in U.S. POINTER, we hypothesized that vascular risk biomarkers, in particular, would be more closely associated with cognition in U.S. POINTER compared to ADNI, potentially contributing to the reduced role of tau in U.S. POINTER.

## 2 Methods

### 2.1 Participants and cohorts

We included baseline data from participants in U.S. POINTER Imaging Ancillary Study and ADNI Study. The U.S. POINTER Imaging Ancillary Study, conducted across 5 U.S. sites, aimed to test whether either of two multidomain lifestyle interventions (low-intensity “self-guided” lifestyle intervention vs. high-intensity “structured” lifestyle intervention) affected AD- and dementia-related brain biomarkers in community-recruited older adults without significant cognitive impairment aged 60–79 years who were randomly assigned in a single-blind design to one of the two intervention arms using electronic medical records and grassroots outreach (Baker et al., 2024). Eligibility criteria included lacking significant memory impairment, being sedentary, reporting a suboptimal diet, and being at risk for future cognitive decline based on factors such as family history of memory impairment, underrepresented ethnoracial group membership, and/or other risk factors (Baker et al., 2024, Baker et al., 2025; Harrison et al., 2025; Landau et al., 2025, Landau et al., 2026; Alzheimer's Association, 2026; Whitmer et al., 2025). ADNI was a longitudinal natural history study of AD conducted across 60+ U.S. and Canadian sites that included a variety of biomarker and clinical assessments and was designed to serve as a model for clinical trials (Weiner et al., 2017). A matched ADNI subsample served as a comparison cohort in the current study because its earlier phases (from which we included participants in this study) excluded participants with vascular comorbidities and, in contrast to U.S. POINTER recruitment, used a convenience sampling approach in academic research centers and memory clinics (Alzheimer's Disease Neuroimaging Initiative (ADNI), 2026; Petersen et al., 2010). Later recruitment in ADNI3 was expanded to include referrals, digital advertising, and an online health registry (Barger et al., 2020). All participants in both cohorts provided written informed consent. This study followed the Strengthening the Reporting of Observational Studies in Epidemiology (STROBE) reporting guidelines.

### 2.2 Cohort matching

To create comparable U.S. POINTER and ADNI samples with similar demographic and clinical characteristics, we first excluded individuals missing concurrent Aβ-PET, tau-PET, and MRI scans. We also excluded ADNI patients with substantial clinical impairment that exceeded the range of clinical impairment in U.S. POINTER (a clinical diagnosis of Alzheimer's disease, a Mini-Mental State Examination (MMSE) score < 23, or a Clinical Dementia Rating (CDR) sum of boxes >2). Propensity score matching was performed using the MatchIt package in R (Zhao et al., 2021), matching ADNI participants with U.S. POINTER participants on age, sex, CDR sum of boxes, and APOE ε4 carrier status. Each ADNI participant could be matched to a U.S. POINTER participant up to two times, resulting in a final matched sample of 775 U.S. POINTER participants and 405 ADNI participants (see Table 1 for cohort characteristics).

**Table 1:** Descriptive statistics and inferential statistics for matched U.S. POINTER and ADNI cohorts.

		U.S. POINTER	ADNI	Matched cohort difference
Demographic	N	775	405	
	Age, years	68.9 +/− 5.2	69.9 +/− 5.4	*p* = .003**
	Female sex, n (%)	487 (62.8%)	235 (58%)	*p* = .12
	Education, years	16 +/− 2.2	16.6 +/− 2.2	*p* < .001***
	URG race, n (%)	249 (32.1%)	111 (27.5%)	*p* = .11
	APOE ε4 carrier, n (%)	246 (31.7%)	135 (33.3%)	*p* = .62
	APOE ε4 genotype, n (%)			*p* = .8
	Non-carrier	529 (68.3%)	270 (66.7%)	
	Heterozygous carrier	220 (28.4%)	119 (29.4%)	
	Homozygous carrier	26 (3.4%)	16 (4%)	
	Glucose	104 +/− 23.1	102.1 +/− 26.8	*p* = .26
	A/G ratio^†^	1.6 +/− 0.3	1.9 +/− 0.3	*p* < .001***
	MCI (%)	46 (5.9%)	94 (23.2%)	p < .001***
Cognition	Global CDR = 0.5, n (%)	171 (22.1%)	84 (20.7%)	*p* = .34
	CDR sum of boxes	0.2 +/− 0.3	0.2 +/− 0.4	*p* = .46
	PACC	−0.02 +/− 0.6	0.2 +/− 0.7	*p* < .001***
	Verbal memory	11.1 +/− 4	12.2 +/− 4.2	*p* < .001***
	Executive function^‡^	−4.4 +/− 0.4	−4.3 +/− 0.4	*p* < .001***
Vascular risk	Systolic blood pressure, mmHg	131.9 +/− 16.1	132.5 +/− 16.1	*p* = .54
	Hypertension (>150 mmHg)	106 (13.7%)	53 (13.7%)	*p = 1*
	BMI	30.2 +/− 5.6	28.1 +/− 5.5	*p* < .001***
	Smokers, n (%)	344 (44.4%)	32 (8.3%)	*p* < .001***
	Diabetes, n (%)	150 (19.4%)	60 (15.5%)	*p* = .13
	FRS	33.4 ± 21.3	23 ± 13.5	p < .001***
Biomarkers	Aβ PET positive, n, (%)	237 (30.6%)	126 (31.1%)	*p* = .90
	Aβ PET Centiloid	18.4 +/− 26.8	19.2 +/− 32.6	*p* = .65
	Entorhinal tau, Z score	0.7 +/− 2.2	0.6 +/− 2.1	*p* = .32
	Meta-temporal tau, Z score	0.3 +/− 1.8	0.7 +/− 2.4	*p* = .01**
	WMH volume (adj)	1.0 +/− 0.7	1.1 +/− 0.8	*p* = .08
	Entorhinal thickness, mm	3.1 +/− 0.2	3.2 +/− 0.3	*p* < .001***
	Hipp volume (adj)	7.3 +/− 0.6	7.3 +/− 0.7	*p* = .10

Abbreviations: URG = Underrepresented Racial Group, ε4 = ε4 allele, MCI = Mild Cognitive Impairment, CDR = Clinical Dementia Rating, PACC = Preclinical Alzheimer Cognitive Composite, BMI = Body Mass Index, FRS = Framingham Risk Scores, PET = Positron Emission Tomography, CL = Centiloid, WMH = White Matter Hyperintensity (log-transformed, intracranial-volume-adjusted WMH volume, unitless), adj = adjusted, Hipp = Hippocampal (intracranial-volume-adjusted hippocampal volume). ** *p* < .01, *** *p* < .001. † A/G (albumin to globulin ratio) was calculated as albumin / (total protein – albumin). A lower A/G ratio reflects a relative decrease in albumin and increase in globulins, indicating greater systemic inflammation and poorer nutritional status(Shibutani et al., 2015). ^‡^ Executive function was assessed using the Trail Making Test Part B (Arbuthnott and Frank, 2000), which was log-transformed and directionally reversed to align with the scaling of other cognitive measures.

### 2.3 Demographic and clinical assessments

Demographic information, including age, sex, education, self-reported ethnoracial group membership (dichotomized as either underrepresented racial/ethnic group (URG) or non-Hispanic White), as well as APOE ε4 genotype, was collected as described previously (Landau et al., 2025, Landau et al., 2026).

Cognitive assessments included global CDR, CDR sum of boxes, the Preclinical Alzheimer's Cognitive Composite (PACC; Papp et al., 2017), which was harmonized for this study using assessments that were available in both cohorts and served as a global cognition measure, and domain-specific components of the PACC that served as verbal memory and executive function scores. The PACC was calculated by averaging z-transformed scores from five cognitive tests. *Z*-scores for each component were standardized using the mean and standard deviation derived from the pooled sample comprising both U.S. POINTER and ADNI participants (Landau et al., 2026). Four cognitive measures were shared across cohorts: Trail Making Test B (log-transformed and reverse-scored to align with other measures, representing executive function; Arbuthnott and Frank, 2000), Mini-Mental State Examination (MMSE; Folstein et al., 1975), Logical Memory Delayed Recall from the Wechsler Memory Scale (verbal memory; Wechsler, 2009), and Category Fluency (animals; Rosen, 1980). The fifth component differed by cohort, with ADNI using the Alzheimer's Disease Assessment Scale (ADAS) word recall (Kueper et al., 2018) and U.S. POINTER using the Free and Cued Selective Reminding Test (FCSRT; Lemos et al., 2015). Higher PACC and domain-specific scores indicate better cognitive performance.

Cardiovascular risk factors (i.e., non-neuroimaging vascular risk measures) included systolic blood pressure, body mass index (BMI), smoking history, and presence of diabetes.

### 2.4 Image acquisition & preprocessing

#### 2.4.1 PET

PET acquisition(Harrison et al., 2023; Landau et al., 2025, Landau et al., 2026), preprocessing (Landau et al., 2024), and analysis methods were harmonized to enable cohort comparisons (Landau et al., 2025). All Aβ-PET images in U.S. POINTER were acquired with [^18^F] Florbetaben (FBB) tracer, while 50% of the matched ADNI sample used FBB (50% matched ADNI) and the other 50% used [^18^F] Florbetapir (FBP). For tau-PET images, U.S. POINTER used [^18^F] MK6240, whereas ADNI used [^18^F] Flortaucipir (FTP).

Standardized uptake value ratios (SUVRs) for Aβ were calculated using the FreeSurfer-defined whole cerebellum as a reference region and a FreeSurfer-defined global cortical summary region as the target region of interest (Landau et al., 2025), then converted to Centiloid (CL) units using tracer-specific equations. Aβ positivity thresholds were set at 18 CL for FBB and 20 CL for FBP, consistent with thresholds derived from our processing pipeline (Landau et al., 2026). Tau SUVRs were calculated using an inferior cerebellar gray matter reference region, defined using the SUIT template and reverse-normalized to each subject's native space to minimize off-target binding in the dorsal cerebellum (Diedrichsen, 2006; Baker et al., 2017), and FreeSurfer-defined entorhinal cortex and meta-temporal region (entorhinal, amygdala, parahippocampal, fusiform, inferior temporal, and middle temporal regions; Jack Jr. et al., 2017) as the target regions of interest.

For the statistical models that included regional tau-PET measures from both cohorts, we standardized entorhinal and meta-temporal cortex tau SUVRs (FTP in ADNI; MK6240 in U.S. POINTER) using reference groups of cognitively unimpaired, Aβ- (Aβ negative) individuals aged 60–70 years to define region-specific mean and standard deviation for tau PET z-scores as described previously (Landau et al., 2025). For voxel-wise analyses, general linear models (GLMs) were fitted separately within each cohort. Tau-PET images from both cohorts were spatially normalized to the T1w MNI-152 space provided by SPM12 to allow voxel-wise analyses. However, direct voxel-wise comparisons between cohorts were not conducted due to the use of different tau tracers and because our z-normalization approach, which was used to harmonize regional tau measures, could not be applied at the voxel level.

#### 2.4.2 MRI

MRI acquisition and quantification methods were also standardized to enable cohort comparisons (Landau et al., 2025). All POINTER scans were acquired at 5 U.S. sites using 3 T Siemens scanners (5 scanner models); ADNI scans were acquired across 57 imaging centers using 3 T GE, Philips, and Siemens scanners. T1-weighted images in both cohorts used a harmonized 3D MPRAGE protocol (TR = 2300 ms, minimum full TE, 1 mm isotropic resolution). FLAIR images were acquired in 3D at 1 × 1 × 1.2 mm resolution (TR = 4800 ms, TE 300–450 ms, TI 1550–1800 ms), with minor site-level variation. T2*-weighted GRE images used for cerebral microbleed detection were acquired using a 3-echo sequence (TE = 6, 13, 20 ms; TR = 650 ms; 0.9 × 0.9 × 4 mm resolution).

T1-weighted images were preprocessed with FreeSurfer v7.1 to define cortical regions of interest (ROIs) and reference regions in native space for both Aβ- and tau- PET quantification. The regional gray matter volume and cortical thickness measures were then extracted. The primary brain atrophy variables were entorhinal thickness and hippocampal volume.

White matter hyperintensity volumes were quantified using FLAIR and T1-weighted images. Briefly, White matter hyperintensity volume was segmented from T1 and FLAIR images using a Bayesian approach, as previously described (DeCarli et al., 2013; Harrison et al., 2025). White matter hyperintensity quantification was volumetric (i.e., segmented white matter hyperintensity volumes in native space), not a lesion count or radiological rating scale. Cerebral microbleeds were identified from 3-echo T2* GRE images by trained image analysts and confirmed by neuroradiologists masked to participants' clinical information, with anatomical localization determined via atlas-based warping to the T2* GRE image (Harrison et al., 2025).

Volumetric measures were adjusted for intracranial volume (ICV) in the current study by a residual adjusted strategy (Voevodskaya et al., 2014), based on a linear relationship established in a reference cohort of 283 Aβ-, cognitively normal older adults from ADNI (Harrison et al., 2025): 1) adjusted hippocampal volume = unadjusted hippocampal volume - (3.2968 × 10^−3^ x (ICV-1432.55)); 2) adjusted white matter hyperintensity volumes (i.e., segmented white matter hyperintensity volume in native space) = ln(unadjusted white matter hyperintensity volume + 1) - (4.0241 × 10^−4^ x (ICV - 1432.55)). Adjusted white matter hyperintensity volumes are unitless.

### 2.5 Statistical analysis

Following cohort matching, we evaluated differences in demographic and clinical characteristics between the U.S. POINTER and ADNI cohorts. Continuous variables were compared using two-sided independent-samples *t*-tests, and categorical variables were compared using chi-square tests.

#### 2.5.1 Tau–cognition models

To examine cohort differences in the relationship between regional tau burden (i.e., entorhinal and meta-temporal cortex) and cognition, we fitted separate linear models with each tau region, cohort, and their interaction term (tau × cohort) as predictors, and global cognition as the outcome. Analyses were conducted separately for global cognition, verbal memory, and executive function and stratified by Aβ status (Aβ + and Aβ-). All models were adjusted for age (Bernick et al., 2012), sex (Pike, 2017), education (Koepsell et al., 2008), and the average number of prior cognitive test exposures across components, as some ADNI participants completed multiple cognitive assessments before the baseline visit. Finally, hierarchical linear regression analyses were performed to assess whether inclusion of (1) vascular risk or (2) atrophy measures described below altered the tau × cohort interaction on cognitive outcomes.

We carried out voxel-wise whole-brain analyses to explore spatial patterns of association between tau burden and cognitive performance (global cognition, verbal memory, and executive function), stratified by Aβ status and cohort. Generalized linear models (GLMs) were estimated at each voxel, with tau-PET SUVR signal as the dependent variable and each cognitive score entered separately as the independent variable. Models were adjusted for age, sex, education, and the average number of prior cognitive test exposures across cognitive measures. All voxel-wise GLMs were implemented in SPM12 running on MATLAB.

Although this model specification differs superficially from the regional models described above (in which tau burden was entered as the predictor and cognition as the outcome), the two specifications are statistically equivalent for testing the association between tau burden and cognitive performance (Giuliani et al., 2005); we specified tau-PET burden as the dependent variable in the voxel-wise models to conform to standard SPM12 general linear model syntax for whole-brain imaging analyses.

#### 2.5.2 Vascular risk–cognition models

To examine cohort differences in the relationship between vascular risk and cognition, we applied the same linear modeling framework, replacing tau measures with vascular risk biomarkers as predictors. Models were adjusted for the same covariates and stratified by cognitive domains (global cognition, verbal memory, and executive function) and Aβ status (Aβ + and Aβ-). Separate models were fitted for each of four non-neuroimaging vascular risk factors (systolic blood pressure, body mass index, smoking history, or diabetes) and for each of two neuroimaging-based cerebrovascular measures (white matter hyperintensity volumes or cerebral microbleeds), along with cohort and the corresponding interaction term (vascular risk × cohort).

#### 2.5.3 Atrophy–cognition models

Brain atrophy models were conducted with entorhinal cortical thickness and hippocampal volume entered as independent variables in separate models, with entorhinal tau burden included as an additional covariate to assess tau-independent associations with cognition. Hierarchical linear regression models were also performed to assess whether brain atrophy altered the tau × cohort interaction on cognitive outcomes.

False discovery rate (FDR) and Bonferroni family-wise error (FWE) corrections were applied to adjust *p*-values and control for type I error across all primary analyses (corrected *p* < .05). For voxel-wise whole-brain analyses, a more stringent random field theory-based FWE correction was applied to account for multiple comparisons across the whole brain (corrected *p* < .05). Detailed mathematical specifications of all statistical models are provided in Supplementary Methods.

## 3 Results

### 3.1 Demographic characteristics of the matched cohorts

After cohort matching, no significant differences were observed between the matched U.S. POINTER and ADNI cohorts in sex, CDR sum of boxes, or APOE ε4 carrier status (*p* > .05). However, participants in the ADNI cohort were, on average, 0.96 years older than those in U.S. POINTER (*p* < .01). As a result, age was included as a covariate in data analyses to adjust for potential confounding effects.

As summarized in Table 1, compared to matched ADNI participants, U.S. POINTER participants had fewer years of education, higher BMI, a higher proportion of individuals with smoking history, lower meta-temporal tau burden, and lower entorhinal cortical thickness.

The cohorts were matched on CDR sum of boxes scores, and global CDR also did not differ, with the majority of individuals from both cohorts with a global CDR of zero, and similar proportions with a global CDR of 0.5 (ADNI, 20.7%; POINTER, 22.1%). Furthermore, a smaller proportion of POINTER (5.9%) than ADNI (23.2%) participants had a diagnosis of MCI, suggesting a mismatch between CDR and clinical diagnosis between cohorts. Despite similar CDR sum of boxes scores, global CDR scores, and a lower proportion of individuals with an MCI diagnosis, we observed worse performance across all cognitive domains (i.e., global cognition, verbal memory, and executive function) in POINTER than ADNI.

### 3.2 Associations between Tau and cognition by cohort and Aβ status

Among ADNI Aβ + individuals, higher entorhinal tau was significantly associated with poorer performance on global cognition, verbal memory, and executive function (Fig. 1A; all FDR-adjusted *p* < .05 and FWE Bonferroni corrected *p* < .05). In contrast, among U.S. POINTER Aβ + individuals, higher entorhinal tau was significantly associated only with poorer verbal memory (FDR-adjusted *p* < .05), with no significant associations observed for global cognition or executive function. Among Aβ- individuals, no significant associations between entorhinal tau and cognitive performance were found in either cohort. Significant cohort × entorhinal tau interaction effects further indicated stronger associations between entorhinal tau and cognition in ADNI compared to U.S. POINTER across all cognitive domains among Aβ + participants (global cognition, verbal memory, and executive function; all FDR-adjusted *p* < .05, with Bonferroni FWE-corrected significance observed for global cognition and verbal memory *p* < .05). A similar pattern was observed for meta-temporal tau, with stronger tau–cognition associations in ADNI than in U.S. POINTER among Aβ + individuals but not among Aβ- individuals (Fig. 1B). However, the interaction of cohort and meta-temporal tau among Aβ + individuals did not reach statistical significance for verbal memory. The inclusion of vascular/cerebrovascular and brain atrophy measures most closely associated with cognition (e.g., white matter hyperintensity volumes, Table S1; hippocampal volume, Table S2) to the model did not alter the tau × cohort interaction and resulted in only minimal improvement in the explained variance in cognition (ΔR^2^ ≤ 0.02).

**Fig. 1:**
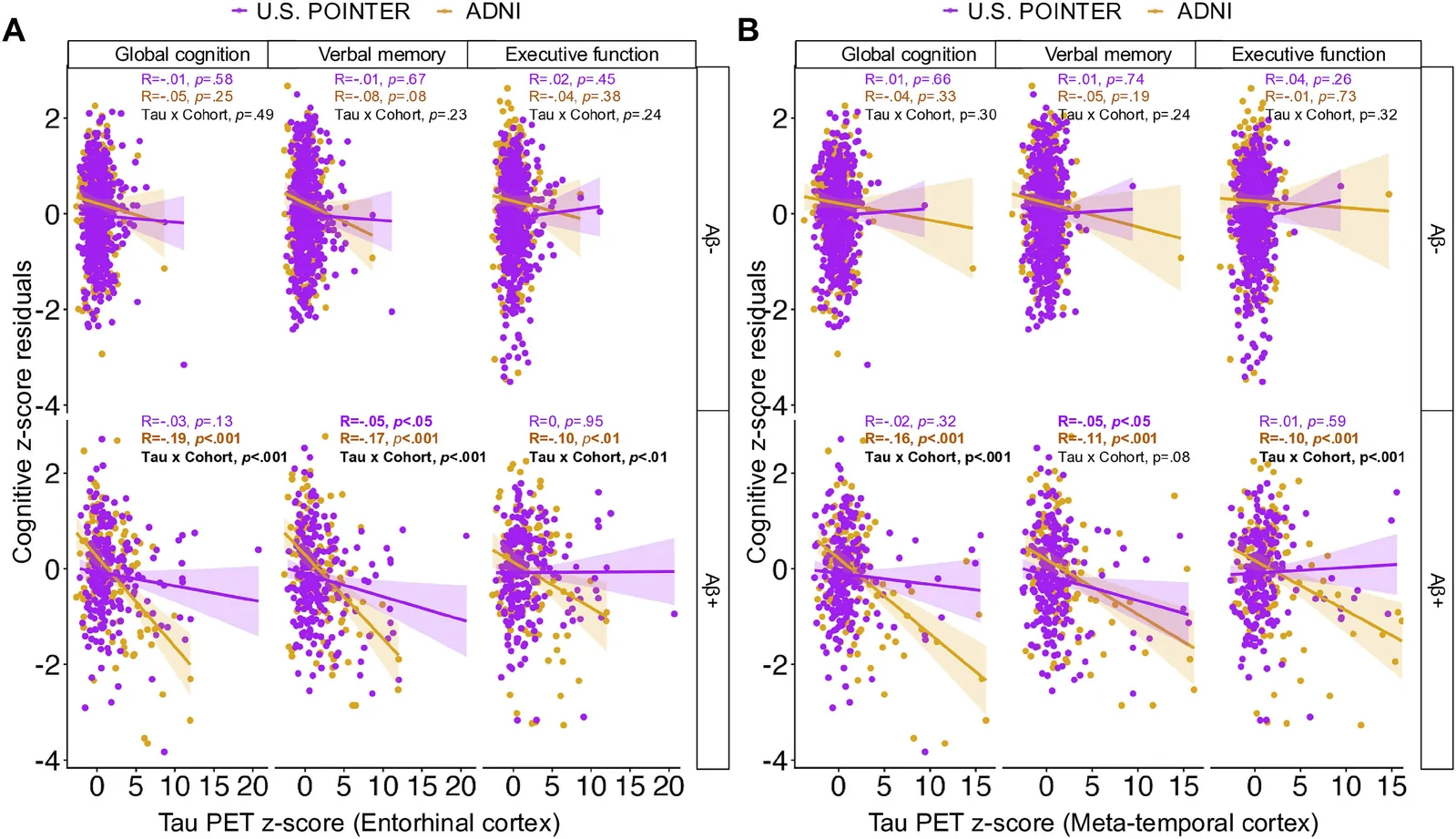
Tau is associated with cognition more strongly in ADNI than POINTER, especially in Aβ + individuals. Scatterplots show the associations between z-scored entorhinal tau-PET (A) and meta-temporal tau-PET (B) with residualized cognitive performance (z-scores) across three domains: global cognition, verbal memory, and executive function, stratified by Aβ status. Residualized cognition represents z-transformed cognitive scores after adjustment for age, sex, education, and the average number of prior cognitive test exposures across cognitive measures. Each data point represents a participant from U.S. POINTER (purple) or ADNI (goldenrod). Cohort-specific regression lines are shown with shaded 95% confidence intervals. Regression coefficients (R) and uncorrected *p*-values are reported by cohort, and statistically significant associations (*p* < .05) are shown in bold.

We next performed exploratory voxel-wise whole-brain analyses among U.S. POINTER and ADNI Aβ + individuals to identify regions, not limited to the entorhinal and meta-temporal ROIs, where tau burden was associated with cognitive performance. Among U.S. POINTER Aβ + individuals, no statistically significant voxel-wise associations were observed between tau burden and global cognition, verbal memory, or executive function (FWE-corrected *p* < .05, cluster size >100 voxels; Fig. 2A). In contrast, among ADNI Aβ + individuals, widespread negative associations were observed between tau burden and cognitive performance (FWE-corrected *p* < .05, cluster size >100 voxels; Fig. 2B). Specifically, poorer global cognition was associated with greater tau burden in the bilateral fusiform gyri, left temporal pole, right parahippocampal gyrus, right inferior temporal gyrus, left olfactory cortex, and right posterior cingulate cortex. Poorer verbal memory was associated with greater tau burden in the left fusiform gyrus, bilateral parahippocampal gyri, and left middle temporal gyrus. Poorer executive function was associated with greater tau burden in the left fusiform gyrus (Table 2).

**Fig. 2:**
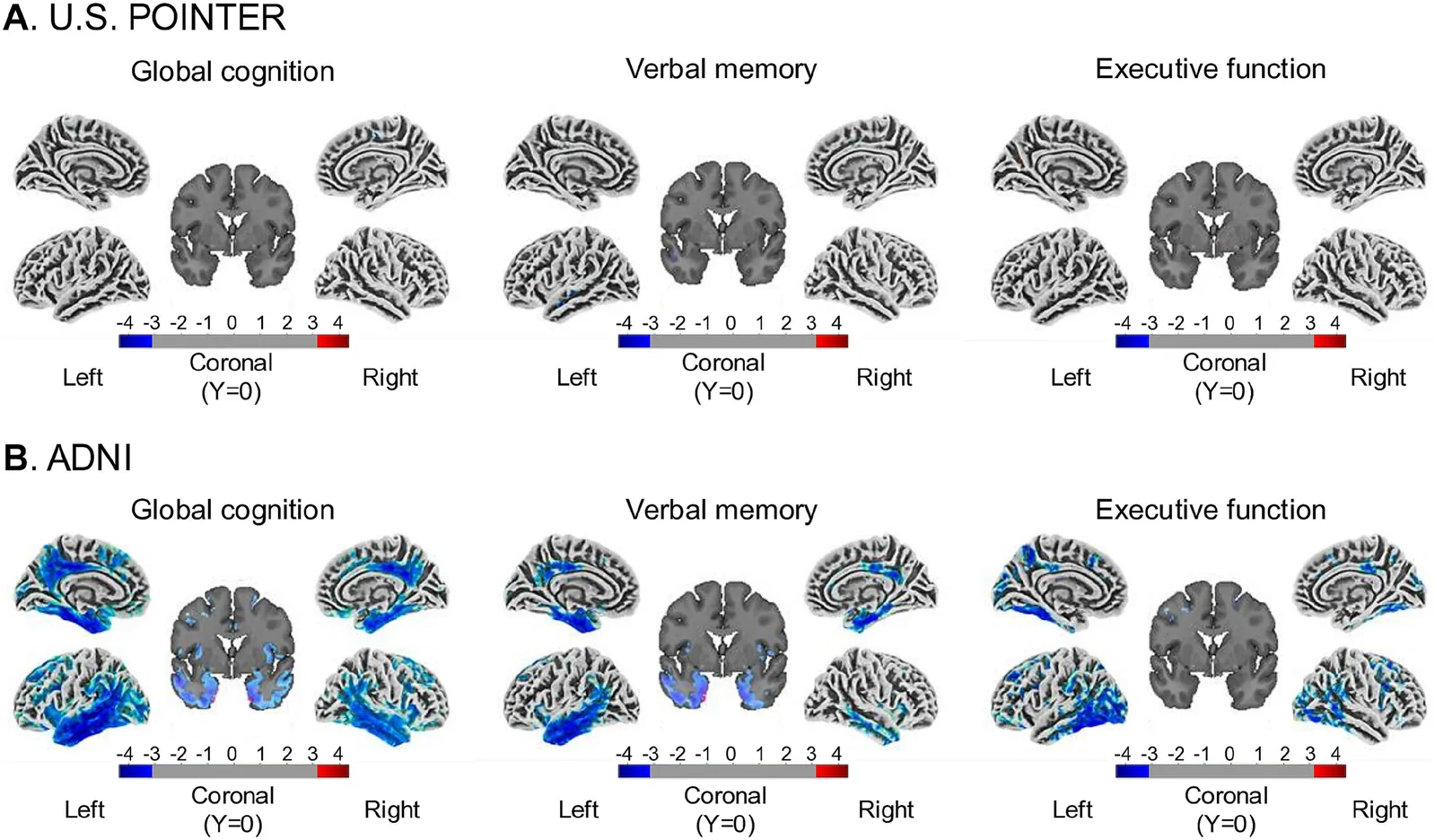
Cognition associates more widely with tau burden in ADNI than POINTER among Aβ + individuals. Statistical t-maps display significant voxel-wise associations (*p* < .001 uncorrected, cluster size >20 voxels) between tau-PET SUVR and cognitive domains among (A) U.S. POINTER (tau tracer: MK6240) and (B) ADNI (tau tracer: FTP) Aβ + individuals. Three brain views are shown: left hemisphere, coronal slice (Y = 0), and right hemisphere. In ADNI Aβ + participants, widespread negative tau–cognition associations were observed in the fusiform gyrus, parahippocampal regions, lateral temporal cortex, and posterior cingulate. No significant clusters were detected in U.S. POINTER Aβ + participants.

**Table 2:** Voxel-wise whole-brain analyses of the associations between tau burden and cognition in the ADNI Aβ + older adults.

Cognition	Peak brain region	Peak t values	Peak MNI coordinates	Number of voxels
Global cognition	Left fusiform gyrus	−7.42	−39, −57, −12	32,413
	Right fusiform gyrus	−6.40	23, −1, −39	968
	Left temporal pole	−6.30	−34, 13, −41	286
	Right parahippocampal gyrus	−6.38	27, −24, −25	1008
	Right inferior temporal gyrus	−5.64	67, −20, −23	137
	Left olfactory cortex	−6.36	−14, 11, −18	116
	Right posterior cingulate cortex	−6.39	3, −48, 27	2802
Verbal memory	Left fusiform gyrus	−5.97	−29, 0, −43	121
	Right parahippocampal gyrus	−6.01	20, −8, −34	298
	Left parahippocampal gyrus	−6.16	−23, −3, −32	671
	Left middle temporal gyrus	−5.71	−60, −3, −19	387
Executive function	Left fusiform gyrus	−5.49	−40, −58, −13	157

Note: Results are FWE-corrected at *p* < .05, cluster size >100 voxels. MNI = Montreal Neurological Institute.

### 3.3 Associations between vascular risk biomarkers and cognition by cohort and Aβ status

Vascular risk biomarkers showed small associations with cognitive performance in U.S. POINTER but not in ADNI (Fig. 3). In ADNI, no significant associations were observed between vascular risk biomarkers and cognition in either Aβ- or Aβ + individuals. In contrast, weak associations were observed in U.S. POINTER among Aβ + participants, although most did not survive FDR correction. Specifically, among Aβ + individuals in U.S. POINTER, better global cognition was associated with lower systolic blood pressure (*p* < .05, uncorrected). Better verbal memory was observed in individuals with higher BMI and in those without cerebral microbleeds (*p* < .05, uncorrected). Better executive function was observed in individuals with the absence of diabetes (FDR-adjusted *p* < .05) and with lower white matter hyperintensity volumes (both FDR-adjusted and Bonferroni FWE-adjusted *p* < .05). Moreover, no significant interaction between vascular risk and cohort survived FDR correction (Table S3), indicating that vascular–cognition associations did not differ significantly between ADNI and U.S. POINTER.

**Fig. 3:**
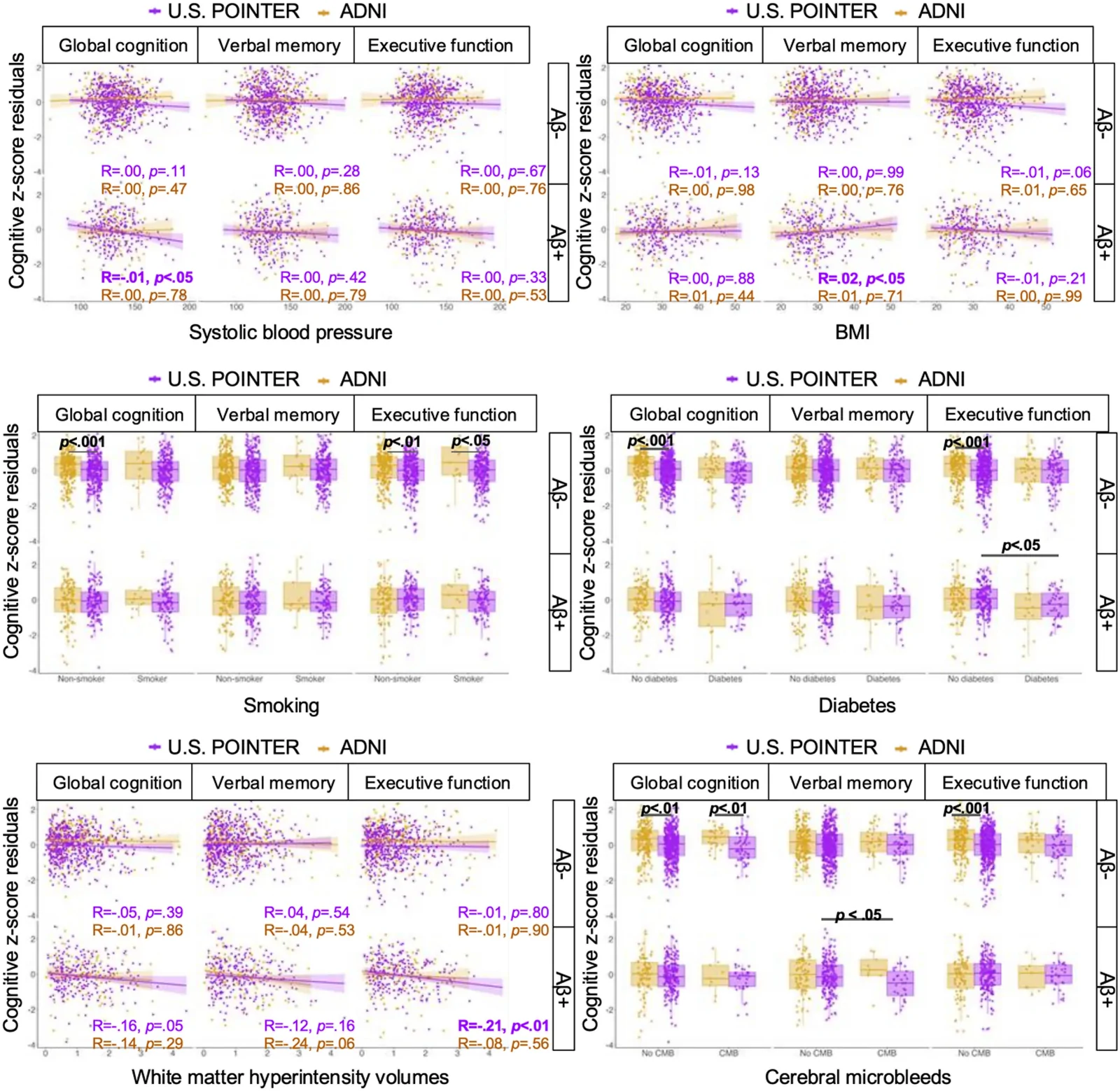
Associations between vascular risk biomarkers and cognition by cohort and Aβ status. Scatter plots illustrate associations between continuous vascular risk biomarkers—systolic blood pressure, BMI, white matter hyperintensity (WMH) volumes, and cerebral microbleeds—and cognitive performance across three domains: global cognition, verbal memory, and executive function. Cognition is represented by z-score transformed residuals after adjusting for age, sex, education, and the average number of prior cognitive test exposures across components. Cohort-specific associations are illustrated using regression lines with shaded 95% confidence intervals. Regression coefficients (R) and uncorrected *p*-values are reported. Statistically significant associations (uncorrected *p* < .05) are denoted in bold. Boxplots illustrate differences in cognition by categorical vascular risk biomarkers (smoking history and diabetes) across cohorts and Aβ status. Individual data points represent participants from the U.S. POINTER (purple) and ADNI (goldenrod) cohorts.

To further explore these findings, we conducted a series of follow-up analyses. First, we tested for three-way interactions involving Aβ status (vascular risk biomarker × cohort × Aβ status). None of these interactions reached statistical significance (all *p* > .05). Second, to assess the composite effects of vascular risk biomarkers on cognition, we calculated each participant's Framingham Risk Score (FRS), a composite index incorporating age, sex, systolic blood pressure, antihypertensive treatment (y/n), BMI, smoking status (y/n), and history of diabetes (y/n; D'Agostino Sr. et al., 2008).

No significant associations were observed between FRS and cognitive performance after adjusting for age, sex, education, and average number of prior cognitive test exposures across cognitive measures (Fig. S1). Third, to quantify the variance in cognitive performance explained by vascular risk biomarkers, we fitted linear regression models stratified by cohort and Aβ status, including all six vascular risk predictors simultaneously and adjusting for age, sex, education, and the average number of prior cognitive test exposures across cognitive measures (Fig. S2&S3). In these models, none of the associations survived the multiple comparison correction (FDR-adjusted *p* > .05), though there were some associations that reached uncorrected statistical significance among Aβ + individuals in U.S. POINTER (*p* < .05, uncorrected). In particular, higher systolic blood pressure was associated with lower global cognition. Higher BMI was associated with better verbal memory. Presence of diabetes was associated with lower verbal memory and lower executive function. Presence of cerebral microbleeds was associated with lower verbal memory. Higher white matter hyperintensity volumes were associated with lower executive function. No significant associations were observed in ADNI or among Aβ- individuals in either cohort.

### 3.4 Associations between brain atrophy and cognition by cohort and Aβ status, controlling for entorhinal Tau burden

We then examined whether regional brain atrophy could account for cohort-specific differences in the tau–cognition relationship. As shown in Fig. 4A, greater entorhinal cortical thickness was associated with better global cognition in both Aβ- and Aβ + ADNI participants and with better verbal memory in Aβ + ADNI participants only (*p* < .05, uncorrected; FDR-adjusted *p* > .05). However, the interaction between cohort and entorhinal cortical thickness was not significant after FDR correction (*p* < .05, uncorrected; FDR-adjusted *p* > .05). As shown in Fig. 4B, greater hippocampal volume was also significantly associated with better global cognition and verbal memory among Aβ + ADNI participants (both FDR-adjusted and Bonferroni FWE-adjusted *p* < .05) and with better executive function among Aβ- ADNI participants (FDR-adjusted *p* < .05). The interaction between cohort and hippocampal volume was significant only for verbal memory among Aβ + individuals (FDR-adjusted *p* < .05). These patterns were further supported by vertex-wise analyses presented in the Supplementary Materials (Fig. S4), which showed broader cortical associations with cognition in the posterior cingulate, temporal pole, and frontal cortex in ADNI compared to U.S. POINTER among Aβ + individuals.

**Fig. 4:**
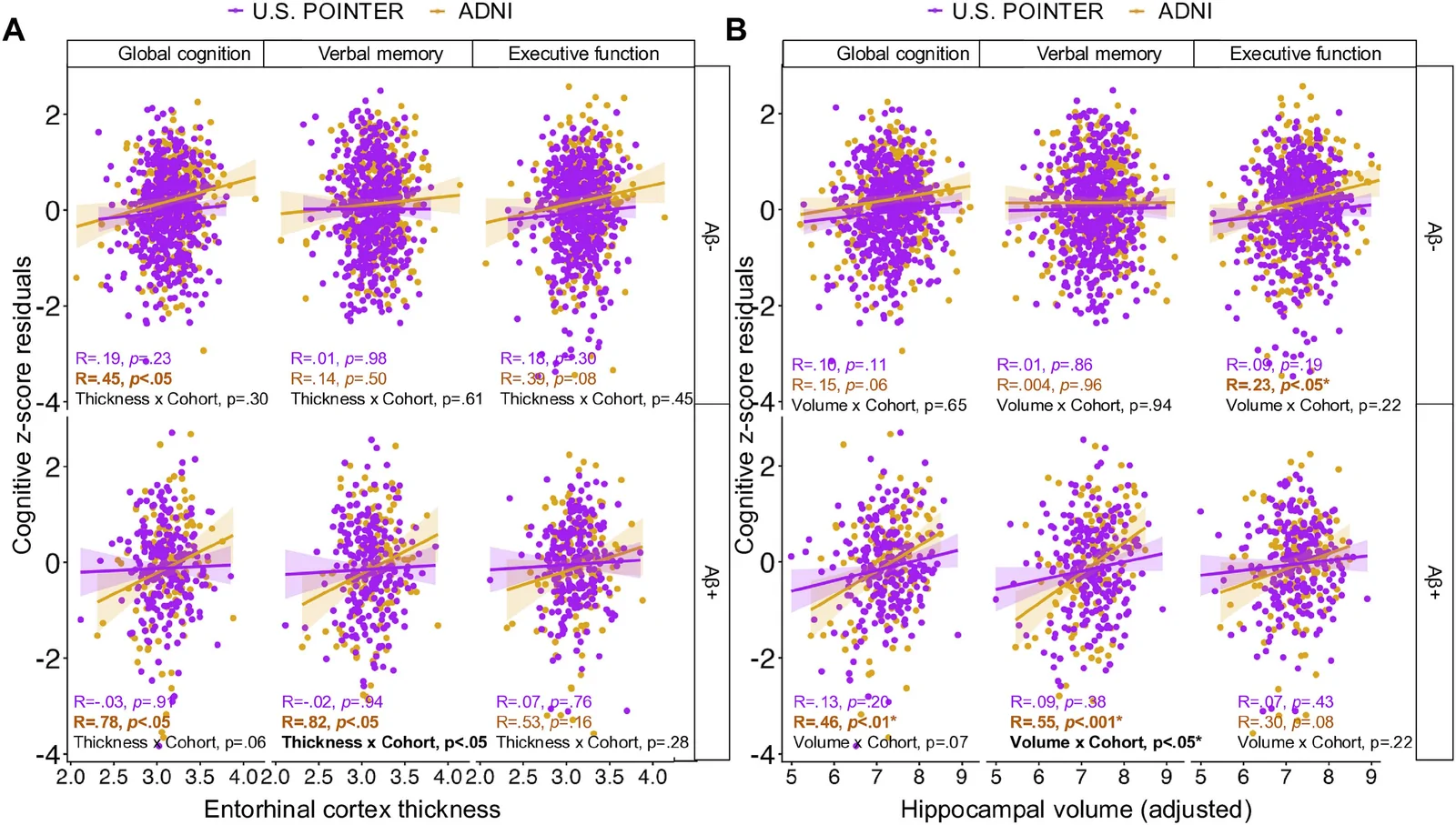
Associations between regional brain atrophy and cognitive performance, stratified by cohort and Aβ status. Scatter plots illustrate associations between brain atrophy ((A) entorhinal cortical thickness and (B) hippocampal volume) and residualized cognitive performance (z-score) across global cognition, verbal memory, and executive function. Regression coefficients (R) and uncorrected *p*-values are reported. Statistically significant associations (uncorrected *p* < .05) are shown in bold. Individual data points represent participants from the U.S. POINTER (purple) and ADNI (goldenrod) cohorts. Asterisks indicate associations that survived FDR-correction (FDR-corrected *p* < .05).

## 4 Discussion

In this study, we examined the roles of variables that have been frequently linked to cognitive performance in older adult cohorts: AD and cerebrovascular neuroimaging biomarkers, measures of brain structure, and vascular risk measures. We compared the roles of these variables in two cohort samples without significant cognitive impairment and matched on age, sex, and clinical characteristics: U.S. POINTER, a community-recruited and vascular risk-enriched cohort, and ADNI, a clinic-based cohort. There were three main findings. First, we extended findings from a previous study showing that associations between tau and global cognition were more robust in ADNI than in U.S. POINTER (Landau et al., 2026) and identified stronger associations between tau and cognitive function across domains (verbal memory, executive function) in ADNI than in U.S. POINTER, particularly among Aβ + older adults. Importantly, the addition of vascular and atrophy measures to the tau models did not meaningfully influence these findings. Further, the independent associations between vascular risk biomarkers and cognition were unexpectedly weak or absent, with associations more frequently observed in U.S. POINTER, although the difference between cohorts did not reach statistical significance. Finally, brain atrophy, particularly hippocampal volume, demonstrated a tau-independent role in contributing to verbal memory in ADNI but not U.S. POINTER, especially Aβ + individuals. Together, these results indicated that matched U.S. POINTER and ADNI samples showed substantial differences in the cross-sectional relationship between AD biomarkers and cognition that could not be accounted for by differences in vascular risk and brain atrophy.

### 4.1 Evaluating potential explanations of cohort differences in biomarker relationships

Our findings from ADNI are consistent with previous reports showing strong cross-sectional associations between temporal tau burden and cognition in cognitively unimpaired individuals (Demirsoy, 2025; Lowe et al., 2019), particularly in the presence of Aβ pathology (Lowe et al., 2019). However, the U.S. POINTER findings contrast with these results, raising questions about the generalizability of AD biomarker–cognition relationships.

A key difference between the cohorts is that the ADNI sample was primarily recruited from academic specialty clinics, whereas the U.S. POINTER sample was primarily recruited using electronic medical records and grassroots community outreach (Hill et al., 2023), suggesting the possibility that AD biomarkers may play a weaker role in cognitive performance in real-world, community-based populations. In addition to being community-based, the U.S. POINTER sample was enriched for health risk characteristics such as elevated blood pressure (Baker et al., 2024, Baker et al., 2025; Harrison et al., 2025; Landau et al., 2025, Landau et al., 2026; Whitmer et al., 2025), which has led us to examine a variety of non-AD biomarkers and health risk variables as potential contributors to cognitive performance.

### 4.2 Vascular risk

We found unexpectedly weak associations between vascular/cardiovascular risk biomarkers and cognition. Differences between cohorts in the role of these vascular risk biomarkers did not reach significance, and interactions between Aβ and vascular risk biomarkers also did not reach significance. However, in analyses of each cohort separately, several vascular–cognition associations were observed among Aβ + individuals in the U.S. POINTER cohort. Specifically, systolic blood pressure showed a weak negative association with global cognition in the present study. Prior systematic review evidence indicates that the negative impact of elevated blood pressure on cognition tends to diminish with advancing age (Forte and Casagrande, 2020), which may partially explain why this association was observed in U.S. POINTER but not in the relatively older ADNI cohort. Counterintuitively, higher BMI was associated with better verbal memory in the present study. This finding may align with prior reports of the ‘obesity paradox,’ where higher BMI in older adults has been linked to better survival and health outcomes since higher BMI may signal better nutritional status and physiological reserve that may confer resilience against age-related cognitive decline (Dramé and Godaert, 2023). Consistent with our findings, previous studies have reported poorer executive function in individuals with diabetes compared to healthy controls (Yeung et al., 2009), and episodic memory has been negatively associated with white matter hyperintensity volumes in U.S. POINTER (Harrison et al., 2025). Previous research supports our findings that the presence of cerebral microbleeds is associated with cognitive impairment (Lei et al., 2013). Cerebral microbleeds are considered markers of small vessel disease and vascular fragility and may reflect cumulative microvascular injury that disrupts distributed neural networks supporting memory and executive function (Akoudad et al., 2016). In addition, although white matter hyperintensity volumes demonstrated the most robust vascular–cognition association among Aβ + older adults, inclusion of this marker in hierarchical regression models did not alter the tau × cohort interaction, statistically suggesting that vascular risk did not account for the observed cohort differences in tau–cognition relationships.

Composite vascular risk, as indexed by the FRS, was not associated with cognition in either U.S. POINTER or ADNI after age, sex, education, and average number of prior cognitive test exposures across components. These findings suggested that, in this cross-sectional context, composite vascular risk had limited independent associations with cognition. Aligning with the generally weak or null vascular–cognition associations observed in these samples, models including multiple vascular risk biomarkers explained minimal variance in cognitive performance across subgroups.

In summary, these findings suggested that vascular risk appeared to play a limited role in explaining cognition in either cohort. A key hypothesis of this study was that the community-based characteristics and recruitment emphasis on vascular risk in the U.S. POINTER participants might have increased the role of vascular risk in explaining cognition, particularly given the reduced role of tau compared to ADNI. But our findings did not support this hypothesis, perhaps because the recruitment criteria in U.S. POINTER may have limited variability in vascular risk, thereby reducing the likelihood of the vascular risk being related to cognitive performance.

### 4.3 Brain atrophy

We also examined cohort differences in the role of brain structure in explaining cognitive performance. Greater entorhinal cortical thickness and hippocampal volume were associated with better cognitive performance across multiple domains—especially verbal memory—in ADNI but not U.S. POINTER, and these relationships in ADNI were stronger in Aβ + than Aβ- individuals, even after adjusting for entorhinal tau burden. We focused on entorhinal cortical thickness and hippocampal volume, reflecting a limbic-predominant AD brain atrophy (Ten Kate et al., 2018; Risacher et al., 2017; Schwarz et al., 2016; Lorenzini et al., 2025), given that our sample consists of individuals without significant cognitive impairment, in whom medial temporal regions are typically the earliest to be affected by AD-related neurodegeneration. These findings were further supported by vertex-wise whole-brain analyses of cortical thickness, which revealed broader cortical associations with cognition—especially verbal memory—in ADNI compared to U.S. POINTER among Aβ + participants, particularly in regions known to be vulnerable to neurodegeneration in AD (Risacher and Saykin, 2013). This provided further evidence for a tau-independent role of brain atrophy more broadly in cognitive performance in ADNI but not POINTER especially in Aβ + individuals.

This result suggested that cognitive performance in ADNI may be more tightly linked to both tau burden and neurodegeneration, whereas cognition in the community-based U.S. POINTER cohort may reflect a more heterogeneous set of contributing influences, including a weak influence of vascular factors alongside other measurements that may be difficult to quantify, including potential resilience mechanisms. Importantly, inclusion of brain atrophy in hierarchical models did not attenuate the tau × cohort interaction, indicating that structural neurodegeneration alone does not account for the observed cohort differences in tau–cognition relationships.

Overall, our findings suggested that vascular and other health risk measures, such as diabetes, small vessel disease markers (e.g., cerebral microbleeds and white matter hyperintensity volume), and brain atrophy, could not explain the poorer baseline cognitive performance in U.S. POINTER. This pattern was consistent across neuropsychological domains, including memory, executive function, and global cognition. Recruitment approaches and other unmeasured comorbidities, risk factors, or aspects of medical history—such as duration of illness, chronic sedentary behavior, or poor diet—may have also contributed to the observed differences, though these were not directly assessed in the current study.

A key strength of this study is the inclusion of a community-based cohort, which enhances the generalizability of established AD biomarker–cognition relationships beyond highly selected clinic-based samples. However, several limitations should be considered. First, although community-based, the U.S. POINTER cohort was selected for elevated vascular risk but largely preserved cognition, which may have restricted variability in the variables and attenuated detectable associations. Second, the multi-site design of U.S. POINTER may introduce site-related heterogeneity, potentially increasing measurement noise and impacting observed associations. Third, the cohorts were matched on variables representing the degree of disease progression, so it is unlikely that the cohorts differed with respect to disease stage, but we note that there was conflicting evidence for the degree of disease progression in each cohort since meta-temporal tau was lower in POINTER than ADNI, but cognitive performance was lower in POINTER, and the cohorts did not differ on APOE4 rates. Finally, these analyses are cross-sectional. Longitudinal data will be necessary to clarify how tau, vascular risk, and brain structure relate to longitudinal changes in cognition.

## 5 Conclusion

Our findings demonstrate that AD pathology is a stronger predictor of cognitive performance in a matched ADNI subsample than in the U.S. POINTER cohort despite ADNI participants exhibiting better overall cognitive performance, and that other biomarkers—such as vascular risk and brain atrophy—offer limited explanatory power for this cohort difference. Although the cohorts were matched on key characteristics relevant to AD biomarker prevalence and cognitive status, they differed in recruitment strategies and enrollment criteria. These differences suggest that AD pathology may influence cognition differently in clinic-based cohorts (e.g., ADNI) compared to community-based samples (e.g., U.S. POINTER), highlighting the importance of considering cohort recruitment methods and sample characteristics when interpreting AD biomarker–cognition relationships. Future longitudinal studies are needed to determine whether predictors of cognitive decline differ between community-based and traditionally clinic-recruited AD cohorts.

## CRediT authorship contribution statement

**Peiwei Liu:** Writing – review & editing, Writing – original draft, Methodology, Formal analysis, Data curation, Conceptualization. **Theresa M. Harrison:** Writing – review & editing, Project administration, Methodology, Data curation, Conceptualization. **Heather M. Snyder:** Writing – review & editing. **Charles DeCarli:** Writing – review & editing. **Pauline Maillard:** Writing – review & editing. **Prashanthi Vemuri:** Writing – review & editing. **Danielle Harvey:** Writing – review & editing. **Robert Koeppe:** Writing – review & editing. **William Jagust:** Writing – review & editing, Supervision, Project administration, Methodology, Data curation, Conceptualization. **Laura D. Baker:** Writing – review & editing, Funding acquisition. **Susan M. Landau:** Writing – review & editing, Visualization, Validation, Supervision, Resources, Project administration, Methodology, Investigation, Funding acquisition, Formal analysis, Data curation, Conceptualization.

## Declaration of competing interest

The authors declare that they have no known competing financial interests or personal relationships that could have appeared to influence the work reported in this paper.

## Data Availability

Data will be made available on request.
