# Supplementary material for: Are the factors associated with cognitive performance generalizable across cohorts?
Source: Neuroimage Clin. 2026 Sep 18;52:104064. doi: 10.1016/j.nicl.2026.104064 (PMC13631531; doi:10.1016/j.nicl.2026.104064)
Supplement: Supplementary file 1 [file mmc1.docx]

**Supplementary material**

Table of Contents

[**Supplementary Methods**	3](#_Toc223287922)

[**Supplementary Results**	4](#_Toc223287923)

[Table S1. Hierarchical linear regression analyses of associations between entorhinal tau and cognitive outcomes and the incremental contribution of white matter hyperintensities among Aβ+ older adults	4](#_Toc223287924)

[Table S2. Hierarchical linear regression analyses of associations between entorhinal tau and cognitive outcomes and the incremental contribution of hippocampal volume among Aβ+ older adults	5](#_Toc223287925)

[Table S3. Associations between vascular risk factors and cognition by cohort and Aβ status	6](#_Toc223287926)

[Figure S1. Associations between Framingham Risk Score (FRS) and cognitive performance across domains, stratified by cohort and Aβ status..	10](#_Toc223287927)

[Figure S2. Estimated regression coefficients (±95% confidence intervals) for associations between individual vascular biomarkers and cognitive domains.	11](#_Toc223287928)

[Figure S3. Unique variance in cognitive performance explained by each vascular biomarker.	12](#_Toc223287930)

[Figure S4. Vertex-wise association between cortical thickness and cognitive performance among Aβ+ older adults in U.S. POINTER (A) and ADNI (B).	13](#_Toc223287932)

[**Appendix 1. ADNI Investigators and Staff (includes authors)**	14](#_Toc223287934)

[**Appendix 2. POINTER Imaging Investigators and Staff (includes authors)**	15](#_Toc223287935)

[**Reference**	36](#_Toc223287936)

**Supplementary Methods**

**Statistical Analysis**

**Tau–cognition models**

$$Cognition_{i}= \beta_{0}+ \beta_{1}Tau_{i}+ \beta_{2}Cohort_{i}+ \beta_{3}\left( Tau_{i}\times Cohort_{i} \right)+ \sum_{k} \beta_{k}C_{ki}+ \varepsilon_{i}$$

where $Cognition_{i}$ represents the cognitive outcome of interest (global cognition, verbal memory, or executive function). Separate models were fitted for each cognitive outcome. $Tau_{i}$ represents either entorhinal cortex tau or meta-temporal tau, entered in separate models; $Cohort_{i}$ is a binary cohort indicator representing ADNI versus U.S. POINTER; $C_{ki}$ represents the $k$-th covariate for participant $i$, including age, sex, education, and the average number of prior cognitive test exposures across components; $\beta_{0}$ is the intercept; $\beta_{1}-\beta_{k}$ are model coefficients and $\varepsilon_{i}$ is the residual error. Analyses were conducted separately within the Aβ+ and Aβ- groups.

**Vascular Risk–Cognition Models**

$$Cognition_{i}=\beta_{0}+\beta_{1}VascularRisk_{i}+\beta_{2}Cohort_{i}+\beta_{3}\left( VascularRisk_{i}\times Cohort_{i} \right)+\sum_{k} \beta_{k}C_{ki}+\varepsilon_{i}$$

where $Cognition_{i}$ represents the cognitive outcome of interest (global cognition, verbal memory, or executive function). Separate models were fitted for each cognitive outcome. $VascularRisk_{i}$ represents individual vascular risk biomarkers, including systolic blood pressure, body mass index, smoking history, diabetes, white matter hyperintensities, and cerebral microbleeds, entered in separate models; $Cohort_{i}$ is a binary cohort indicator representing ADNI versus U.S. POINTER; $C_{ki}$ represents the $k$-th covariate for participant $i$, including age, sex, education, and the average number of prior cognitive test exposures across components; $\beta_{0}$ is the intercept; $\beta_{1}-\beta_{k}$ are model coefficients and $\varepsilon_{i}$ is the residual error. Analyses were conducted separately within the Aβ+ and Aβ- groups.

**Atrophy–Cognition Models**

$$Cognition_{i}=\beta_{0}+\beta_{1}BrainAtrophy_{i}+\beta_{2}Cohort_{i}+\beta_{3}\left( BrainAtrophy_{i}\times Cohort_{i} \right)+\sum_{k} \beta_{k}C_{ki}+\varepsilon_{i}$$

where $Cognition_{i}$ represents the cognitive outcome of interest (global cognition, verbal memory, or executive function). Separate models were fitted for each cognitive outcome. $BrainAtrophy_{i}$ represents entorhinal cortical thickness or hippocampal volume, entered in separate models; $Cohort_{i}$ is a binary cohort indicator representing ADNI versus U.S. POINTER; $C_{ki}$ represents the $k$-th covariate for participant $i$, including age, sex, education, and the average number of prior cognitive test exposures across components; $\beta_{0}$ is the intercept; $\beta_{1}-\beta_{k}$ are model coefficients and $\varepsilon_{i}$ is the residual error. Analyses were conducted separately within the Aβ+ and Aβ- groups.

**Vertex-wise whole-brain analysis (Figure S4)** was conducted to examine associations between cortical thickness and cognitive performance (global cognition, verbal memory, and executive function) in U.S. POINTER and ADNI, particularly among Aβ+ older adults. T1-weighted MRI scans were preprocessed using the Computational Anatomy Toolbox (CAT12) in MATLAB to generate vertex-wise estimates of cortical thickness^1^. Generalized linear models (GLMs) were run at each vertex, with cortical thickness as the dependent variable and each cognitive domain analyzed separately as the independent variable. All models were adjusted for age, sex, education, average prior test exposure, and entorhinal tau SUVR. Analyses were performed within CAT12 in MATLAB.

**Supplementary Results**

**Table S1. Hierarchical linear regression analyses of associations between entorhinal tau and cognitive outcomes and the incremental contribution of white matter hyperintensities among Aβ+ older adults**

|  | Predictor | Model 1 (coefficient) | *p* value | Model 2 (coefficient) | *p* value |
| --- | --- | --- | --- | --- | --- |
| Global cognition | Tau | -0.18 | <0.001 | -0.19 | <0.001 |
|  | Cohort | -0.40 | 0.004 | -0.43 | 0.05 |
|  | Tau x cohort | 0.15 | <0.001 | 0.15 | <0.001 |
|  | WMH | - |  | -0.19 | 0.09 |
|  | WMH x cohort | - |  | 0.02 | 0.86 |
|  | Model R^2^ (adj.) | 0.20 |  | 0.21 |  |
|  | ΔR^2^ |  |  | 0.01 |  |
| Verbal memory | Tau | -0.72 | <0.001 | -0.74 | <0.001 |
|  | Cohort | -1.44 | 0.01 | -2.21 | 0.01 |
|  | Tau x cohort | 0.53 | <0.001 | 0.54 | <0.001 |
|  | WMH | - |  | -1.19 | 0.01 |
|  | WMH x cohort | - |  | 0.63 | 0.27 |
|  | Model R^2^ (adj.) | 0.15 |  | 0.17 |  |
|  | ΔR^2^ |  |  | 0.02 |  |
| Executive function | Tau | -0.04 | 0.004 | -0.04 | 0.003 |
|  | Cohort | -0.07 | 0.23 | -0.02 | 0.85 |
|  | Tau x cohort | 0.04 | 0.02 | 0.04 | 0.02 |
|  | WMH | - |  | -0.05 | 0.31 |
|  | WMH x cohort | - |  | -0.04 | 0.46 |
|  | Model R^2^ (adj.) | 0.07 |  | 0.08 |  |
|  | ΔR^2^ |  |  | 0.01 |  |

Note. Model 1 and Model 2 have been adjusted by age, sex, education, and average number of prior cognitive test exposures across components. WMH = white matter hyperintensities

**Table S2. Hierarchical linear regression analyses of associations between entorhinal tau and cognitive outcomes and the incremental contribution of hippocampal volume among Aβ+ older adults**

|  | Predictor | Model 1 (coefficient) | *p* value | Model 2 (coefficient) | *p* value |
| --- | --- | --- | --- | --- | --- |
| Global cognition | Tau | -0.18 | <0.001 | -0.17 | <0.001 |
|  | Cohort | -0.40 | 0.004 | 0.69 | 0.61 |
|  | Tau x cohort | 0.15 | <0.001 | 0.15 | <0.001 |
|  | Hipp vol. | - |  | 0.34 | 0.03 |
|  | Hipp vol. x cohort | - |  | -0.15 | 0.43 |
|  | Model R^2^ (adj.) | 0.20 |  | 0.22 |  |
|  | ΔR^2^ |  |  | 0.01 |  |
| Verbal memory | Tau | -0.18 | <0.001 | -0.15 | <0.001 |
|  | Cohort | -0.35 | 0.01 | 2.05 | 0.14 |
|  | Tau x cohort | 0.13 | <0.001 | 0.11 | 0.004 |
|  | Hipp vol. | - |  | 0.45 | 0.003 |
|  | Hipp vol. x cohort | - |  | -0.32 | 0.08 |
|  | Model R^2^ (adj.) | 0.15 |  | 0.17 |  |
|  | ΔR^2^ |  |  | 0.02 |  |
| Executive function | Tau | -0.09 | 0.004 | -0.08 | 0.02 |
|  | Cohort | -0.17 | 0.23 | 0.69 | 0.62 |
|  | Tau x cohort | 0.09 | 0.02 | 0.09 | 0.03 |
|  | Hipp vol. | - |  | 0.23 | 0.13 |
|  | Hipp vol. x cohort | - |  | 0.09 | 0.03 |
|  | Model R^2^ (adj.) | 0.07 |  | 0.07 |  |
|  | ΔR^2^ |  |  | 0.003 |  |

Note. Model 1 and Model 2 have been adjusted by age, sex, education, and average number of prior cognitive test exposures across components. Hipp vol. = hippocampal volume

**Table S3. Associations between vascular risk factors and cognition by cohort and Aβ status**

| Cognition (Outcome) | Aβ status | Terms | Statistic | p value (uncorr.) | p value (FDR corr.) |
| --- | --- | --- | --- | --- | --- |
| Global cognition | Aβ- | Systolic BP | 0.71 | 0.40 | 0.53 |
|  |  | Cohort | 16.09 | **<0.001** | **<0.001** |
|  |  | Systolic BP x Cohort | 2.33 | 0.13 | 0.38 |
|  | Aβ+ | Systolic BP | 3.08 | 0.08 | 0.48 |
|  |  | Cohort | 0.06 | 0.81 | 0.81 |
|  |  | Systolic BP x Cohort | 2.74 | 0.10 | 0.38 |
| Verbal memory | Aβ- | Systolic BP | 0.63 | 0.43 | 0.53 |
|  |  | Cohort | 2.39 | 0.12 | 0.25 |
|  |  | Systolic BP x Cohort | 0.60 | 0.44 | 0.88 |
|  | Aβ+ | Systolic BP | 0.60 | 0.44 | 0.53 |
|  |  | Cohort | 0.12 | 0.73 | 0.81 |
|  |  | Systolic BP x Cohort | 0.04 | 0.84 | 0.93 |
| Executive function | Aβ- | Systolic BP | 0.01 | 0.90 | 0.90 |
|  |  | Cohort | 13.36 | **<0.001** | **<0.001** |
|  |  | Systolic BP x Cohort | 0.24 | 0.62 | 0.93 |
|  | Aβ+ | Systolic BP | 1.37 | 0.24 | 0.53 |
|  |  | Cohort | 0.32 | 0.57 | 0.81 |
|  |  | Systolic BP x Cohort | 0.01 | 0.93 | 0.93 |
| Global cognition | Aβ- | BMI | 4.13 | **0.04** | 0.13 |
|  |  | Cohort | 13.39 | **<0.001** | **0.01** |
|  |  | BMI x Cohort | 0.72 | 0.40 | 0.65 |
|  | Aβ+ | BMI | 0.37 | 0.54 | 0.65 |
|  |  | Cohort | 0.06 | 0.81 | 0.81 |
|  |  | BMI x Cohort | 0.38 | 0.54 | 0.65 |
| Verbal memory | Aβ- | BMI | 0.02 | 0.88 | 0.88 |
|  |  | Cohort | 2.36 | 0.12 | 0.25 |
|  |  | BMI x Cohort | 0.05 | 0.81 | 0.81 |
|  | Aβ+ | BMI | 3.06 | 0.08 | 0.16 |
|  |  | Cohort | 0.27 | 0.61 | 0.73 |
|  |  | BMI x Cohort | 0.66 | 0.42 | 0.65 |
| Executive function | Aβ- | BMI | 4.06 | **0.04** | 0.13 |
|  |  | Cohort | 11.01 | **<0.001** | **0.01** |
|  |  | BMI x Cohort | 2.01 | 0.16 | 0.65 |
|  | Aβ+ | BMI | 0.68 | 0.41 | 0.62 |
|  |  | Cohort | 0.49 | 0.48 | 0.72 |
|  |  | BMI x Cohort | 0.47 | 0.49 | 0.65 |
| Global cognition | Aβ- | Smoking | 1.06 | 0.30 | 0.96 |
|  |  | Cohort | 15.24 | **<0.001** | **<0.001** |
|  |  | Smoking x Cohort | 0.04 | 0.84 | 0.84 |
|  | Aβ+ | Smoking | 0.03 | 0.87 | 0.96 |
|  |  | Cohort | 0.02 | 0.90 | 0.90 |
|  |  | Smoking x Cohort | 3.49 | 0.06 | 0.38 |
| Verbal memory | Aβ- | Smoking | 0.01 | 0.92 | 0.96 |
|  |  | Cohort | 2.94 | 0.09 | 0.17 |
|  |  | Smoking x Cohort | 0.33 | 0.57 | 0.68 |
|  | Aβ+ | Smoking | 0.31 | 0.58 | 0.96 |
|  |  | Cohort | 0.26 | 0.61 | 0.73 |
|  |  | Smoking x Cohort | 1.04 | 0.31 | 0.46 |
| Executive function | Aβ- | Smoking | 0.92 | 0.34 | 0.96 |
|  |  | Cohort | 12.72 | **<0.001** | **0.01** |
|  |  | Smoking x Cohort | 1.33 | 0.25 | 0.46 |
|  | Aβ+ | Smoking | 0.003 | 0.96 | 0.96 |
|  |  | Cohort | 0.43 | 0.51 | 0.73 |
|  |  | Smoking x Cohort | 1.99 | 0.16 | 0.46 |
| Global cognition | Aβ- | Diabetes | 2.48 | 0.12 | 0.21 |
|  |  | Cohort | 15.57 | **<0.001** | **<0.001** |
|  |  | Diabetes x Cohort | 0.04 | 0.84 | 0.93 |
|  | Aβ+ | Diabetes | 3.11 | 0.08 | 0.21 |
|  |  | Cohort | 0.002 | 0.96 | 0.96 |
|  |  | Diabetes x Cohort | 0.07 | 0.79 | 0.93 |
| Verbal memory | Aβ- | Diabetes | 0.08 | 0.78 | 0.78 |
|  |  | Cohort | 2.32 | 0.13 | 0.26 |
|  |  | Diabetes x Cohort | 0.01 | 0.93 | 0.93 |
|  | Aβ+ | Diabetes | 1.57 | 0.21 | 0.25 |
|  |  | Cohort | 0.05 | 0.82 | 0.96 |
|  |  | Diabetes x Cohort | 0.01 | 0.91 | 0.93 |
| Executive function | Aβ- | Diabetes | 2.20 | 0.14 | 0.21 |
|  |  | Cohort | 13.02 | **<0.001** | **<0.001** |
|  |  | Diabetes x Cohort | 0.18 | 0.67 | 0.93 |
|  | Aβ+ | Diabetes | 7.49 | **0.01** | **0.04** |
|  |  | Cohort | 0.66 | 0.42 | 0.63 |
|  |  | Diabetes x Cohort | 0.16 | 0.69 | 0.93 |
| Global cognition | Aβ- | WMH | 0.28 | 0.60 | 0.89 |
|  |  | Cohort | 18.60 | **<0.001** | **<0.001** |
|  |  | WMH x Cohort | 0.16 | 0.69 | 0.95 |
|  | Aβ+ | WMH | 4.67 | **0.03** | 0.06 |
|  |  | Cohort | 0.45 | 0.51 | 0.61 |
|  |  | WMH x Cohort | 0.02 | 0.88 | 0.95 |
| Verbal memory | Aβ- | WMH | 0.04 | 0.84 | 0.95 |
|  |  | Cohort | 3.74 | 0.05 | 0.11 |
|  |  | WMH x Cohort | 0.75 | 0.39 | 0.78 |
|  | Aβ+ | WMH | 5.19 | **0.02** | 0.06 |
|  |  | Cohort | 0.45 | 0.50 | 0.61 |
|  |  | WMH x Cohort | 0.78 | 0.38 | 0.78 |
| Executive function | Aβ- | WMH | 0.004 | 0.95 | 0.95 |
|  |  | Cohort | 15.22 | **<0.001** | **<0.001** |
|  |  | WMH x Cohort | 0.004 | 0.95 | 0.95 |
|  | Aβ+ | WMH | 6.33 | **0.01** | 0.06 |
|  |  | Cohort | 0.01 | 0.92 | 0.92 |
|  |  | WMH x Cohort | 0.96 | 0.33 | 0.78 |
| Global cognition | Aβ- | CMB | 0.33 | 0.56 | 0.91 |
|  |  | Cohort | 23.51 | **<0.001** | **<0.001** |
|  |  | CMB x Cohort | 3.08 | 0.08 | 0.24 |
|  | Aβ+ | CMB | 1.46 | 0.23 | 0.68 |
|  |  | Cohort | 0.44 | 0.51 | 0.57 |
|  |  | CMB x Cohort | 0.16 | 0.69 | 0.98 |
| Verbal memory | Aβ- | CMB | 0.01 | 0.91 | 0.91 |
|  |  | Cohort | 8.43 | **<0.01** | **<0.01** |
|  |  | CMB x Cohort | 0.04 | 0.84 | 0.98 |
|  | Aβ+ | CMB | 3.23 | 0.07 | 0.44 |
|  |  | Cohort | 2.52 | 0.11 | 0.17 |
|  |  | CMB x Cohort | 3.60 | 0.06 | 0.24 |
| Executive function | Aβ- | CMB | 0.02 | 0.89 | 0.91 |
|  |  | Cohort | 19.50 | **<0.001** | **<0.001** |
|  |  | CMB x Cohort | 0.001 | 0.98 | 0.98 |
|  | Aβ+ | CMB | 0.04 | 0.83 | 0.91 |
|  |  | Cohort | 0.33 | 0.57 | 0.57 |
|  |  | CMB x Cohort | 0.22 | 0.64 | 0.98 |

Abbreviations: BP = Blood Pressure, BMI = Body Mass Index, WMH = White Matter Hyperintensities, CMB = Cerebral Microbleeds

**Table S4.**

Sensitivity Analysis: Associations of Tau Burden, Vascular Risk, and Brain Atrophy with Cognitive Performance by Cohort and Aβ Status, Adjusted for APOE ε4 Dosage.

| **Panel A: Tau–Cognition Models (Entorhinal Cortex Tau)** | | | |
| --- | --- | --- | --- |
|  | **Global Cognition** | **Verbal Memory** | **Executive Function** |
|  | ***Estimate (p-value)*** | ***Estimate (p-value)*** | ***Estimate (p-value)*** |
| **Aβ+ Individuals** | | | |
| U.S. POINTER | -0.01 (p = 0.57) | -0.01 (p = 0.77) | 0.03 (p = 0.38) |
| ADNI | -0.05 (p = 0.25) | -0.08 (p = 0.07) | -0.04 (p = 0.38) |
| ***Cohort × ERC Tau Interaction*** | 0.03 (p = 0.49) | 0.07 (p = 0.19) | 0.07 (p = 0.22) |
| **Aβ− Individuals** | | | |
| U.S. POINTER | -0.02 (p = 0.20) | **-0.04 (p = 0.03)** | 0.00 (p = 0.79) |
| ADNI | **-0.18 (p < .001)*** | **-0.17 (p < .001)*** | **-0.09 (p = 0.01)*** |
| ***Cohort × ERC Tau Interaction*** | **0.16 (p < .001)*** | **0.12 (p < .001)*** | **0.09 (p = 0.01)*** |
| **Panel B: Vascular Risk–Cognition Models (WMH)** | | | |
|  | **Global Cognition** | **Verbal Memory** | **Executive Function** |
|  | ***Estimate (p-value)*** | ***Estimate (p-value)*** | ***Estimate (p-value)*** |
| **Aβ+ Individuals** | | | |
| U.S. POINTER | -0.04 (p = 0.42) | 0.04 (p = 0.53) | -0.01 (p = 0.81) |
| ADNI | -0.02 (p = 0.81) | -0.05 (p = 0.47) | -0.01 (p = 0.86) |
| ***Cohort × WMH Interaction*** | -0.03 (p = 0.75) | 0.09 (p = 0.35) | 0.00 (p = 0.99) |
| **Aβ− Individuals** | | | |
| U.S. POINTER | **-0.18 (p = 0.02)** | -0.14 (p = 0.09) | **-0.23 (p < 0.01)*** |
| ADNI | -0.11 (p = 0.41) | -0.22 (p = 0.09) | -0.06 (p = 0.65) |
| ***Cohort × WMH Interaction*** | -0.07 (p = 0.62) | 0.08 (p = 0.57) | -0.17 (p = 0.23) |
| **Panel C: Brain Atrophy–Cognition Models (Hippocampal Volume)** | | | |
|  | **Global Cognition** | **Verbal Memory** | **Executive Function** |
|  | ***Estimate (p-value)*** | ***Estimate (p-value)*** | ***Estimate (p-value)*** |
| **Aβ+ Individuals** | | | |
| U.S. POINTER | 0.09 (p = 0.15) | 0.00 (p = 0.98) | 0.08 (p = 0.23) |
| ADNI | 0.15 (p = 0.06) | 0.00 (p = 0.98) | **0.23 (p = 0.01)*** |
| ***Cohort × Hipp. Volume Interaction*** | -0.05 (p = 0.59) | 0.00 (p = 1.00) | -0.15 (p = 0.20) |
| **Aβ− Individuals** | | | |
| U.S. POINTER | 0.14 (p = 0.17) | 0.10 (p = 0.33) | 0.08 (p = 0.39) |
| ADNI | **0.48 (p < 0.01)*** | **0.58 (p < .001)*** | 0.32 (p = 0.06) |
| ***Cohort × Hipp. Volume Interaction*** | -0.35 (p = 0.05) | **-0.48 (p = 0.01)*** | -0.24 (p = 0.19) |

Note. All models adjusted for APOE ε4 dosage (0, 1, or 2 alleles) as an additional covariate. p-values are uncorrected unless otherwise indicated.

* FDR-adjusted p < .05.

ERC = entorhinal cortex; WMH = white matter hyperintensities; Hipp. = hippocampal; Aβ+ = amyloid-positive; Aβ− = amyloid-negative.

**Figure S1. Associations between Framingham Risk Score (FRS) and cognitive performance across domains, stratified by cohort and Aβ status.**
**(A)** Linear regression models examined the associations between FRS and cognitive performance, adjusted for education and average prior test exposure. **(B)** Models additionally adjusted for age and sex. Each data point represents an individual from either the U.S. POINTER (purple) or ADNI (goldenrod) cohort. Shaded regions around regression lines represent 95% confidence intervals. Regression coefficients (R) and uncorrected *p*-values are reported by cohort. Statistically significant associations (uncorrected *p* < .05) are shown in bold.

**Figure S2. Estimated regression coefficients (±95% confidence intervals) for associations between individual vascular biomarkers and cognitive domains.**

Linear regression models were fitted separately for each cognitive domain, including all six vascular risk predictors simultaneously and adjusting for age, sex, education, and average number of prior cognitive test exposures. Models were run separately by cohort and stratified by amyloid status (Aβ- and Aβ+). Abbreviations: CMB = cerebral microbleeds; WMH = white matter hyperintensities; BP = blood pressure; BMI = body mass index; CI = confidence interval.

**Figure S3. Unique variance in cognitive performance explained by each vascular biomarker.**

Forest plots display the unique variance (ΔR²) contributed by each biomarker, defined as the change in explained variance between the full regression model and a reduced model excluding that predictor. This unique variance reflects the proportion of variance in each cognitive outcome uniquely attributable to an individual cardiovascular risk factor after adjusting for all other predictors and covariates, including age, sex, education, and average exposure to cognitive tests. Abbreviations: bp = blood pressure, BMI = body mass index, WHM = white matter hyperintensities, CMB = cerebral microbleeds.

**Figure S4. Vertex-wise association between cortical thickness and cognitive performance among Aβ+ older adults in U.S. POINTER (A) and ADNI (B).**

Statistical t-maps display significant voxel-wise associations between cortical thickness and cognitive performance after adjustment for age, sex, education, the average number of prior cognitive test exposures, and entorhinal tau burden. Significance was defined as *p* < .001 (uncorrected) with a cluster size > 20 vertices. Color bars indicate t-statistics.

**Appendix 1. ADNI Investigators and Staff (includes authors)**

| **Name** | **Location** | **Role** | **Contribution** |
| --- | --- | --- | --- |
| Michael W. Weiner, MD | University of California, San Francisco | Principal Investigator | PI |
| John Q. Trojanowski,  MD, PhD | University of Pennsylvania | Core Leader | Coordinated Biomarker Core |
| Leslie Shaw,  PhD | University of Pennsylvania | Core Leader | Coordinated Biomarker Core |
| Laurel Beckett, PhD | University of California, Davis | Core Leader | Coordinated Biostatistics Core |
| Paul Aisen,  MD | University of Southern California | Core Leader | Coordinated Clinical Core |
| Ronald Petersen MD, PhD | Mayo Clinic | Core Leader | Coordinated Clinical Core |
| Andrew J. Saykin, PsyD | Indiana University | Core Leader | Coordinated Genetics Core |
| Arthur W. Toga, PhD | University of Southern California | Core Leader | Coordinated Informatics Core |
| Clifford Jack,  MD | Mayo Clinic, Rochester, Minnesota | Core Leader | Coordinated MRI core |
| John C. Morris, MD | Washington University | Core Leader | Coordinated neuropathology Core |
| William Jagust, MD | University of California, Berkeley | Core Leader | Coordinated PET Core |

**Appendix 2. POINTER Imaging Investigators and Staff (includes authors)**

| **Site** | **First Name** | **Last Name** | **Degree** | **Institution** | **Location** | **Role** | **Active** |
| --- | --- | --- | --- | --- | --- | --- | --- |
| ChicagoLand - Advocate | Darren | Gitelman | MD | Advocate Lutheran General Hospital | Park Ridge, IL | Imaging Site Principal Investigator | Yes |
| ChicagoLand - Advocate | Grace | Lucenti | BS | Advocate Lutheran General Hospital | Park Ridge, Il | Imaging Site Coordinator | Yes |
| ChicagoLand - Advocate | Masun | Jackson | MS | Advocate Lutheran General Hospital | Park Ridge, IL | Imaging Site Project Manager | Yes |
| ChicagoLand - Advocate | Anthony | McCormack | MD | Advocate Lutheran General Hospital | Park Ridge, IL | Site Clinician | Yes |
| ChicagoLand - Advocate | Jestine | Chakkumcal |  | Advocate Lutheran General Hospital | Park Ridge, IL | MRI Technician | Yes |
| ChicagoLand - Advocate | Aniket | Joshi |  | Advocate Lutheran General Hospital | Park Ridge, IL | PET Technician | Yes |
| ChicagoLand - Advocate | Maneesh | Phillip |  | Advocate Lutheran General Hospital | Park Ridge, IL | PET Technician | Yes |
| ChicagoLand - Advocate | Salman | Khan |  | Advocate Lutheran General Hospital | Park Ridge, IL | PET Technician | Yes |
| ChicagoLand - Advocate | Peter | Bilik |  | Advocate Lutheran General Hospital | Park Ridge, IL | PET Technician | Yes |
| ChicagoLand - Advocate | Maha | Haroon | BS | Advocate Lutheran General Hospital | Park Ridge, IL | Research Assistant | Yes |
| ChicagoLand - Advocate | Susan | Panek |  | Advocate Lutheran General Hospital | Park Ridge, IL | Imaging Site Coordinator | No |
| ChicagoLand - Rush | Neelum | Aggarwal | MD | Rush University Medical Center | Chicago, IL | Imaging Site Principal Investigator | Yes |
| ChicagoLand - Rush | Christy | Tangney | PhD | Rush University Medical Center | Chicago, IL | Imaging Site Principal Investigator | Yes |
| ChicagoLand - Rush | Kira | Cella |  | Rush University Medical Center | Chicago, IL | Imaging Site Coordinator / Project Manager | Yes |
| ChicagoLand - Rush | Thomas | Holland | MD | Rush University Medical Center | Chicago, IL | Site Clinician | Yes |
| ChicagoLand - Rush | Ronnie | Daniel |  | Rush University Medical Center | Chicago, IL | MRI Technician | Yes |
| ChicagoLand - Rush | Greg | Lamonica |  | Rush University Medical Center | Chicago, IL | PET Technician | Yes |
| ChicagoLand - Rush | Bethany | Franz | MHA | Rush University Medical Center | Chicago, IL | Imaging Site Coordinator / Project Manager | No |
| ChicagoLand - Rush | Martha | Silva |  | Rush University Medical Center | Chicago, IL | PET Technician | No |
| ChicagoLand - Rush | Amara | Ballard |  | Rush University Medical Center | Chicago, IL | PET Technician | No |
| ChicagoLand - Rush | Karolina | Lukasiak |  | Rush University Medical Center | Chicago, IL | PET Technician | No |
| ChicagoLand - Rush | Jessica | Monteon |  | Rush University Medical Center | Chicago, IL | PET Technician | No |
| ChicagoLand - Rush | Meron | Halile |  | Rush University Medical Center | Chicago, IL | PET Technician | No |
| ChicagoLand - Rush | Daniel | Somers |  | Rush University Medical Center | Chicago, IL | PET Technician | No |
| ChicagoLand - Rush | Kaitlin | Koncilja |  | Rush University Medical Center | Chicago, IL | Imaging Site Coordinator / Project Manager | No |
| ChicagoLand - Rush | Meera | Sotor | MPH | Rush University Medical Center | Chicago, IL | Site Project Manager | No |
| Coordinating Center | Laura | Baker | PhD | Wake Forest University School of Medicine | Winston-Salem, NC | Imaging Study Coordinating Center Principal Investigator | Yes |
| Coordinating Center | Shannon | Risacher | PhD | Wake Forest University School of Medicine | Winston-Salem, NC | Imaging Study Coordinating Center Co-Investigator | Yes |
| Coordinating Center | Marc | Rudolph | PhD | Wake Forest University School of Medicine | Winston-Salem, NC | Imaging Study Coordinating Center Principal Investigator | Yes |
| Coordinating Center | Iris | Leng | PhD | Wake Forest University School of Medicine | Winston-Salem, NC | Imaging Study Coordinating Center Co-Investigator | Yes |
| Coordinating Center | Benjamin | Williams | MD | Wake Forest University School of Medicine | Winston-Salem, NC | US POINTER Safety Committee Chair | Yes |
| Coordinating Center | Nancy | Woolard |  | Wake Forest University School of Medicine | Winston-Salem, NC | Senior Clinical Research Manager | Yes |
| Coordinating Center | Sharon | Wilmoth |  | Wake Forest University School of Medicine | Winston-Salem, NC | Associate Director Clinical Research | Yes |
| Coordinating Center | Leslie | Gordineer |  | Wake Forest University School of Medicine | Winston-Salem, NC | Imaging Study Coordinating Center Project Manager | Yes |
| Coordinating Center | Stephanie | Okonmah-Obazee |  | Wake Forest University School of Medicine | Winston-Salem, NC | Imaging Study Coordinating Center Assistant Project Manager | No |
| Coordinating Center | Christopher | Whitlow | MD, PhD | Wake Forest University School of Medicine | Winston-Salem, NC | Imaging Study Coordinating Center Co-Investigator | No |
| Coordinating Center | Samuel | Lockhart | PhD | Wake Forest University School of Medicine | Winston-Salem, NC | Imaging Study Coordinating Center Principal Investigator | No |
| Coordinating Center Data Core | Mark | Espeland | PhD | Wake Forest University School of Medicine | Winston-Salem, NC | Data CC Director | Yes |
| Coordinating Center Data Core | Laura | Lovato | MS | Wake Forest University School of Medicine | Winston-Salem, NC | Data CC Co-Director / Data Analysis | Yes |
| Coordinating Center Data Core | Robert | Amoroso | BS | Wake Forest University School of Medicine | Winston-Salem, NC | Data CC Co-Director / Programmer | Yes |
| Coordinating Center Data Core | Debbie | Felton | BS | Wake Forest University School of Medicine | Winston-Salem, NC | Data CC Safety Project Manager | Yes |
| Coordinating Center Data Core | John | Nichols | BS | Wake Forest University School of Medicine | Winston-Salem, NC | Analyst / Programmer IV | Yes |
| Coordinating Center Data Core | Danielle | Harvey | PhD | University of California, Davis | Sacramento, California | Biostatistician | Yes |
| Coordinating Center Data Core | Katie | Garcia | MS | Wake Forest University School of Medicine | Winston-Salem, NC | Biostatistician | Yes |
| Coordinating Center Data Core | Majorie | Howard | MS | Wake Forest University School of Medicine | Winston-Salem, NC | Biostatistician | Yes |
| Coordinating Center Data Core | Julissa | Almomte Santana | BS | Wake Forest University School of Medicine | Winston-Salem, NC | Project Manager | Yes |
| Coordinating Center Data Core | Tara | Beckner | BS | Wake Forest University School of Medicine | Winston-Salem, NC | Project Manager | Yes |
| Coordinating Center Data Core | Jennifer | Talton | MS | Wake Forest University School of Medicine | Winston-Salem, NC | Senior Biostatistician | Yes |
| Coordinating Center Data Core | Kaylla | Richardson | MS | Wake Forest University School of Medicine | Winston-Salem, NC | Statistical Analyst / Programmer | Yes |
| Coordinating Center Data Core | Jennifer | Walker |  | Wake Forest University School of Medicine | Winston-Salem, NC | Data CC Project Manager | No |
| Coordinating Center Data Core | Julia | Spell | BS | Wake Forest University School of Medicine | Winston-Salem, NC | Data CC Co-Director | No |
| Houston | Valory | Pavlik | PhD | Baylor College of Medicine | Houston, Texas | Site Principal Investigator | Yes |
| Houston | Joseph | Masdeu | MD, PhD | Houston Methodist | Houston, Texas | Imaging Site Principal Investigator | Yes |
| Houston | Melissa | Yu | MD | Baylor College of Medicine | Houston, Texas | Site Co-Investigator | Yes |
| Houston | Shayla | Yonce |  | Baylor College of Medicine | Houston, Texas | Site Manager | Yes |
| Houston | Sunny | Chen | BA | Houston Methodist | Houston, Texas | Imaging Site Coordinator | Yes |
| Houston | Maria | Mora |  | Houston Methodist | Houston, Texas | Imaging Site Co-Coordinator | Yes |
| Houston | Juan | Toledo | MD, PhD | Houston Methodist | Houston, Texas | Imaging Staff | Yes |
| Houston | Vi | Phan |  | Houston Methodist | Houston, Texas | MRI Technician | Yes |
| Houston | Hong | Nguyen |  | Houston Methodist | Houston, Texas | MRI Technician | Yes |
| Houston | Andrea | Altenbaugh |  | Houston Methodist | Houston, Texas | MRI Technician | Yes |
| Houston | Lien | My Phan |  | Houston Methodist | Houston, Texas | MRI Technician / Imaging Staff | Yes |
| Houston | Kim | Doan |  | Houston Methodist | Houston, Texas | PET Technician | Yes |
| Houston | Jeffrey | Bishop | MD | Baylor College of Medicine | Houston, Texas | Site Clinician | Yes |
| Houston | Asa | McCoy |  | Houston Methodist | Houston, Texas | PET Technician | Yes |
| Houston | Elsa | Ruiz |  | Houston Methodist | Houston, Texas | Imaging Site Co-Coordinator | No |
| Houston | David | De Leon |  | Houston Methodist | Houston, Texas | Imaging Site Co-Coordinator | No |
| Houston | Denna | Tascon |  | Houston Methodist | Houston, Texas | Imaging Site Co-Coordinator | No |
| Houston | Carlos | Zarate |  | Houston Methodist | Houston, Texas | Imaging Site Co-Coordinator | No |
| Houston | Stephanie | Wenzlawsh |  | Houston Methodist | Houston, Texas | Imaging Site Coordinator | No |
| Houston | Brandi | Sanchez |  | Houston Methodist | Houston, Texas | PET Technician | No |
| Houston | Elizabeth | Lipscomb | RN | Baylor College of Medicine | Houston, Texas | Site Manager | No |
| Imaging MRI Core | Charles | DeCarli | MD | University of California - Davis | Sacramento, CA | MRI Core Lead / Analysis | Yes |
| Imaging MRI Core | Prashanthi | Vemuri | PhD | Mayo Clinic | Rochester, MN | Acquisition / QC / Analysis | Yes |
| Imaging MRI Core | Clifford | Jack | MD | Mayo Clinic | Rochester, MN | Acquisition / QC / Analysis | Yes |
| Imaging MRI Core | Youngkyoo | Jung | PhD | University of California - Davis | Sacramento, CA | Analysis | Yes |
| Imaging MRI Core | Pauline | Maillard | PhD | University of California - Davis | Sacramento, CA | Analysis | Yes |
| Imaging MRI Core | Calvin | Reyes |  | Mayo Clinic | Rochester, MN | Analysis | Yes |
| Imaging MRI Core | Denise | Reyes |  | Mayo Clinic | Rochester, MN | Acquisition / QC / Analysis | Yes |
| Imaging MRI Core | Bret | Borowski |  | Mayo Clinic | Rochester, MN | Acquisition | Yes |
| Imaging MRI Core | Ashritha | Reddy |  | Mayo Clinic | Rochester, MN | QC / Analysis | Yes |
| Imaging MRI Core | Leonard | Matoush |  | Mayo Clinic | Rochester, MN | Acquisition | Yes |
| Imaging MRI Core | Samantha | Zuk |  | Mayo Clinic | Rochester, MN | QC / Analysis | Yes |
| Imaging MRI Core | Kally | Gleichner |  | Mayo Clinic | Rochester, MN | Acquisition | Yes |
| Imaging MRI Core | John | Moore-Weiss | PhD | Mayo Clinic | Rochester, MN | Analysis | Yes |
| Imaging MRI Core | Cory | Johnson |  | Mayo Clinic | Rochester, MN | Acquisition | Yes |
| Imaging MRI Core | Kaely | Thostenson |  | Mayo Clinic | Rochester, MN | Acquisition | Yes |
| Imaging MRI Core | Chloe | Pallex |  | Mayo Clinic | Rochester, MN |  | No |
| Imaging MRI Core | Niroja | Harinesan |  | Mayo Clinic | Rochester, MN |  | No |
| Imaging MRI Core | Kohl | Johnson-Sparrman |  | Mayo Clinic | Rochester, MN |  | No |
| Imaging PET Core | Susan | Landau | PhD | University of California, Berkeley | Berkeley, CA | POINTER Imaging Study Principal Investigator | Yes |
| Imaging PET Core | Robert | Koeppe | PhD | University of Michigan Medical School | Ann Arbor, MI | PET Acquisition / QC | Yes |
| Imaging PET Core | Wendy | Liu |  | University of Michigan Medical School | Ann Arbor, MI | PET Acquisition / QC | Yes |
| Imaging PET Core | Guie | Gu |  | University of Michigan Medical School | Ann Arbor, MI | PET Acquisition / QC | Yes |
| Imaging PET Core | Theresa | Harrison | PhD | University of California, Berkeley | Berkeley, CA | PET Core Analysis | Yes |
| Imaging PET Core | William | Jagust | MD | University of California, Berkeley | Berkeley, CA | PET Core Analysis | Yes |
| Imaging PET Core | Tyler | Ward |  | University of California, Berkeley | Berkeley, CA | Research Assistant | Yes |
| Imaging PET Core | Peiwei | Liu |  | University of California, Berkeley | Berkeley, CA | Research Assistant | Yes |
| Imaging PET Core | Jacinda | Taggett |  | University of California, Berkeley | Berkeley, CA | Research Assistant | No |
| Imaging PET Core | Katherine | Kudriavtsev |  | University of California, Berkeley | Berkeley, CA |  | Yes |
| New England - Rhode Island | Hwamee | Oh | Ph.D. | Brown University | Providence, RI | Imaging Site Principal Investigator | Yes |
| New England - Rhode Island | Stephen | Salloway | MD, MS | Butler Hospital | Providence, RI | Site Principal Investigator | Yes |
| New England - Rhode Island | Edward | Huey | MD | Butler Hospital | Providence, RI | Site Principal Investigator | Yes |
| New England - Rhode Island | Marycia | Malloy | BS | Butler Hospital |  | Imaging Site Study Coordinator | Yes |
| New England - Rhode Island | Susan | Bross |  |  |  | MRI Technician, Imaging Staff | Yes |
| New England - Rhode Island | Lidia | Pacheco | AS | Rhode Island Medical Imaging | Pawtucket , RI | MRI Technician, Imaging Staff | Yes |
| New England - Rhode Island | Kerrin | Jackson |  |  |  | PET Technician | Yes |
| New England - Rhode Island | Andrew | Olivier |  |  |  | PET Technician | Yes |
| New England - Rhode Island | Ava | Tower |  |  |  | Imaging Staff, PET Technician | Yes |
| New England - Rhode Island | Netasha | Rocha |  |  |  | Site Supervisor | Yes |
| New England - Rhode Island | Taylor | Morrison |  |  |  | PET leave Technician | Yes |
| New England - Rhode Island | Meghan | Riddle | MD | Butler Hospital | Providence, RI | Imaging Site Co-Investigator | No |
| New England - Rhode Island | Sophia | Tarro |  | Butler Hospital | Providence, RI | Imaging Site Study Coordinator | No |
| New England - Rhode Island | Linda | Davidge |  |  |  | PET Technician | No |
| New England - Rhode Island | Jocelyn | Falardeau |  |  |  | MRI Technician, Imaging Staff | No |
| New England - Rhode Island | Samuel | Slezak |  | Butler Hospital | Providence, RI | Site Manager | No |
| New England - Rhode Island | Eliza | Rego |  | Butler Hospital | Providence, RI | Imaging Site Study Coordinator | No |
| New England - Rhode Island | Caitlin | McManus |  | Butler Hospital | Providence, RI | Imaging Site Study Coordinator | No |
| New England - Rhode Island | Tyler | Rosenholm |  | Butler Hospital | Providence, RI | Site Manager | No |
| New England - Rhode Island | Brandon | Wood |  |  |  | PET Patient Coordinator |  |
| New England - Rhode Island | Rachel | Achilli |  |  |  | PET Patient Coordinator |  |
| New England - Rhode Island | Gina | DeMello | RT(R)(M)(MR)(MRSO | |  | MRI |  |
| New England - Rhode Island | Melissa | Jette | RT(R)(M)(BD) | |  | MRI Site Manager |  |
| New England - Rhode Island | Shelby | Bawden |  |  |  | POINTER Research Coordinator | |
| New England - Rhode Island | Franchesca | Rolon |  |  |  | Pointer Senior Research Assistant | |
| North Carolina - Wake Forest | Maryjo | Cleveland | MD | Wake Forest University School of Medicine | Winston-Salem, NC | Imaging Site Principal Investigator | Yes |
| North Carolina - Wake Forest | Leslie | Teague | MS | Wake Forest University School of Medicine | Winston-Salem, NC | Imaging Site Project Manager | Yes |
| North Carolina - Wake Forest | Rebecca | Davis |  | Wake Forest University School of Medicine | Winston-Salem, NC | Imaging Site Coordinator | Yes |
| North Carolina - Wake Forest | Beth | Lovette |  | Wake Forest University School of Medicine | Winston-Salem, NC | Imaging Staff | Yes |
| North Carolina - Wake Forest | Abbie | Eaton | PhD, MMS | Wake Forest University School of Medicine | Winston-Salem, NC | Site Clinician | Yes |
| North Carolina - Wake Forest | Mary | Ellenburg |  | Wake Forest University School of Medicine | Winston-Salem, NC | Imaging Staff | Yes |
| North Carolina - Wake Forest | Joseph | Bottoms |  | Wake Forest University School of Medicine | Winston-Salem, NC | Imaging Staff / PET Technician | Yes |
| North Carolina - Wake Forest | Lena | Moretz |  | Wake Forest University School of Medicine | Winston-Salem, NC | MRI Technician | Yes |
| North Carolina - Wake Forest | Wendy | Baker |  | Wake Forest University School of Medicine | Winston-Salem, NC | MRI Technician | Yes |
| North Carolina - Wake Forest | Michael | Shannon |  | Wake Forest University School of Medicine | Winston-Salem, NC | MRI Research Technologist | Yes |
| North Carolina - Wake Forest | Philicia | Armstrong |  | Wake Forest University School of Medicine | Winston-Salem, NC | Imaging Site Coordinator | No |
| North Carolina - Wake Forest | Melissa | Ramirez |  | Wake Forest University School of Medicine | Winston-Salem, NC | Imaging Site Coordinator | No |
| North Carolina - Wake Forest | Megan | Lipford | PhD | Wake Forest University School of Medicine | Winston-Salem, NC | Imaging Site Co-Investigator | No |
| North Carolina - Wake Forest | Samuel | Lockhart | PhD | Wake Forest University School of Medicine | Winston-Salem, NC | Imaging Site Principal Investigator | No |
| North Carolina - Wake Forest | Ezequiel | Zamora | MD | Wake Forest University School of Medicine | Winston-Salem, NC | Imaging Site Principal Investigator | No |
| North Carolina - Wake Forest | Jeff | Williamson | MD, MHS | Wake Forest University School of Medicine | Winston-Salem, NC | Cilincal Site PI | No |
| North Carolina - Wake Forest | Christiana | Higgs | MS | Wake Forest University School of Medicine | Winston-Salem, NC | Imaging Staff | No |
| North Carolina - Wake Forest | Benita | Bowman |  | Wake Forest University School of Medicine | Winston-Salem, NC | Imaging Staff | No |
| North Carolina - Wake Forest | Sandra | Kaminski |  | Wake Forest University School of Medicine | Winston-Salem, NC | MRI Technician | No |
| North Carolina - Wake Forest | Freda | Crawford |  | Wake Forest University School of Medicine | Winston-Salem, NC | PET Technician | No |
| North Carolina - Wake Forest | Jonathan | Richardson |  | Wake Forest University School of Medicine | Winston-Salem, NC | PET Technician | No |
| Northern California - UC Davis | Charles | DeCarli | MD | University of California, Davis | Sacramento, California | Imaging Principal Investigator | Yes |
| Northern California - UC Davis | Pauline | Maillard | PhD | University of California, Davis | Sacramento, California | Imaging Co-Investigator | Yes |
| Northern California - UC Davis | Rachel | Whitmer | PhD | University of California, Davis | Sacramento, California | Site Principal Investigator | Yes |
| Northern California - UC Davis | Sarah | Farias | PhD | University of California, Davis | Sacramento, California | Site Principal Investigator | Yes |
| Northern California - UC Davis | Anna | Garzon | MHA | University of California, Davis | Sacramento, California | Site Manager | Yes |
| Northern California - UC Davis | Welver | Suarez | Bachelor's of Science | University of California, Davis | Sacramento, California | Imaging Site Study Coordinator, MRI Technician | Yes |
| Northern California - UC Davis | Tyler | McConnell | Bachelor's of Science | University of California, Davis | Sacramento, California | Imaging Site Project Manager | Yes |
| Northern California - UC Davis | Tessa | Hafenstein | Bachelor's of Science | University of California, Davis | Sacramento, California | Imaging Site Study Coordinator, MRI Technician | Yes |
| Northern California - UC Davis | Parvaneh | Gerami | MSN, CNRN, FNP-BC | University of California, Davis | Sacramento, California | Site Clinician | Yes |
| Northern California - UC Davis | Roberto | Ramos | N/A | University of California, Davis | Sacramento, California | Site Project Manager | No |
| Northern California - UC Davis | Maria | Gallegos | N/A | University of California, Davis | Sacramento, California | MRI Technician | Yes |
| Northern California - UC Davis | Heather | Russell | N/A | Northern California PET Imaging Center | Sacramento, California | PET Technician | Yes |
| Northern California - UC Davis | Ellen | Thomas | N/A | Northern California PET Imaging Center | Sacramento, California | PET Technician | Yes |
| Northern California - UC Davis | Richard | Isip | N/A | Northern California PET Imaging Center | Sacramento, California | PET Technician | Yes |
| Northern California - UC Davis | Hitesh | Patel | N/A | Northern California PET Imaging Center | Sacramento, California | PET Technician | Yes |
| Northern California - UC Davis | Tuan | Pham | N/A | Northern California PET Imaging Center | Sacramento, California | PET Technician | Yes |
| Northern California - UC Davis | Justin | Salts | N/A | University of California, Davis | Sacramento, California | Imaging Site Study Coordinator, MRI Safety Monitor | Yes |
| Northern California - UC Davis | Quynh | Tran | N/A | University of California, Davis | Sacramento, California | MRI Safety Monitor | Yes |
| Northern California - UC Davis | Nicole | Ibewiro | N/A | University of California, Davis | Sacramento, California | MRI Safety Monitor | Yes |
| Northern California - UC Davis | Lyndsey | Guerra | N/A | University of California, Davis | Sacramento, California | MRI Safety Monitor | Yes |
| Northern California - UC Davis | Macaria | Mendoza | N/A | University of California, Davis | Sacramento, California | MRI Safety Monitor | Yes |
| Northern California - UC Davis | Casey | Castro | N/A | University of California, Davis | Sacramento, California | MRI Safety Monitor | No |
| Northern California - UC Davis | Magaly | Quinteros | N/A | University of California, Davis | Sacramento, California | MRI Safety Monitor | No |
| Northern California - UC Davis | Anika | Mehra | N/A | University of California, Davis | Sacramento, California | MRI Safety Monitor | Yes |

**Reference**

1.	Gaser, C. *et al.* CAT: a computational anatomy toolbox for the analysis of structural MRI data. *Gigascience* **13**, giae049 (2024).
